# Longitudinal associations between PM_2.5_ with gestational diabetes mellitus mediated by gut microbiome and potential mechanism: based on a prospective pregnant women cohort in China

**DOI:** 10.3389/fcimb.2026.1749504

**Published:** 2026-02-27

**Authors:** Shanshan Mei, Jingyi Ye, Yaoyao Teng, Yisheng Chen, Yan Long, Xueqin Zhao, Xueqing Cen, Xiaoyan Zhang, Chunyan Zhu

**Affiliations:** 1 Department of Obstetrics, Guangzhou Women and Children’s Medical Centre, Guangzhou Medical University, Guangzhou, China; 2 Department of Epidemiology and Health Statistics, School of Public Health, Guangzhou Medical University, Guangzhou, China; 3 Department of Microbiological Laboratory, Chongqing Jiulongpo District Center for Disease Control and Prevention, Chongqing, China; 4 Department of Disease Prevention and Control, Dachong Community Health Service Center, Zhongshan, China; 5 Liuzhou Hospital, Guangzhou Women and Children’s Medical Center, Guangzhou Medical University, Liuzhou, China; 6 Department of Laboratory, Guangzhou Women and Children’s Medical Centre, Guangzhou Medical University, Guangzhou, China

**Keywords:** blood glucose, circRNAs, gestational diabetes mellitus, gut microbiota, metabolites, PM_2.5_

## Abstract

**Background:**

Exposure to particulate matter pollution with aerodynamic diameters < 2.5 μm (PM_2.5_) has been linked to gestational diabetes mellitus (GDM) and gut microbiota dysbiosis. However, few studies have illustrated the associations among PM_2.5_ exposure, gut microbiota, blood metabolites, circular RNAs (circRNAs) and GDM risk. This study aimed to explore the moderating effects of the gut microbiota on the association between PM_2.5_ exposure and GDM, and to analyze the interaction network of PM_2.5_ exposure, gut microbiota, blood metabolites and circRNAs.

**Methods:**

Participants (n = 1,248) were selected from the Pregnancy Metabolic Disease and Adverse Pregnancy Outcome (PMDAPO) cohort in Guangzhou, China. Demographic information, blood and fecal samples were collected from the participants. The fecal microbial composition and relative abundance were characterized using 16S rRNA gene sequencing, while blood differential metabolites and circRNAs of pregnant women with GDM were assessed using non-targeted metabolomics and RT-qPCR, respectively. Exposure levels of air pollutants were assessed using data from the nearest monitoring station. Spearman correlation and regression models were conducted to estimate the associations among PM_2.5_ exposure, gut microbiota, blood metabolites, circRNAs and GDM.

**Results:**

Elevated PM_2.5_ exposure levels were significantly associated with an increased risk of GDM, impaired glucose homeostasis and gut microbiota dysbiosis. *Solobacterium* and *Escherichia_Shigella* showed a positive effect modification on the association between PM_2.5_ exposure and fasting blood glucose, while *Fusicatenibacter*, *Ruminococcaceae_UBA1819*, *Raoultibacter*, *Anaerofustis* and *Phascolarctobacterium* showed a negative effect modification on the association between PM_2.5_ exposure and 2-h OGTT glucose. GDM-associated gut microbiota, including *Catabacter*, *Angelakisella*, *Romboutsia* and *Fusicatenibacter*, were associated with both GDM-associated metabolites (such as sphinganine-1-phosphate, sphingomyelin) and GDM-associated circRNAs (such as hsa_circ_0006732 and hsa_circ_0001439), which were involved in glycerophospholipid metabolism, sphingolipid metabolism and insulin signaling pathway.

**Conclusions:**

The gut microbiota may moderate the associations between PM_2.5_ exposure and blood glucose levels, and both PM_2.5_ exposure and gut microbiota may be related to GDM, potentially involving pathways such as glycerophospholipid metabolism, sphingolipid metabolism and the insulin signaling pathway. However, lifestyle factors (diet and physical activity) and residential mobility were not measured, and the fecal microbiota was assessed at a single time point in mid-pregnancy. Thus, these limitations may contribute to residual confounding, exposure misclassification, and limited causal inference.

## Introduction

Gestational diabetes mellitus (GDM) is defined as any degree of glucose intolerance that is first recognized during pregnancy, encompassing both previously undiagnosed pregestational diabetes and impaired glucose tolerance occurring during pregnancy, with a rising prevalence (American Diabetes Association Professional Practice Committee, 2022). GDM is highly associated with adverse perinatal pregnancy outcomes. Studies have shown that GDM is associated with an increased risk of maternal complications, including preeclampsia, cesarean delivery, dystocia and postpartum type 2 diabetes mellitus (T2DM) (Bellamy et al., 2009; Ovesen et al., 2015). Offspring of mothers with GDM are at increased risk for preterm birth, congenital anomalies, stillbirth, macrosomia, large-for-gestational-age infants, neonatal hypoglycemia, neonatal respiratory distress syndrome, and hyperbilirubinemia (Ferrara et al., 2007; Mitanchez, 2010; Wielandt et al., 2015). Furthermore, there is a significantly increased risk of obesity, cardiovascular disease, autism, neurodevelopmental disorders, and metabolic syndrome in the offspring (Moon and Jang, 2022). Risk factors for GDM include overweight or obesity, advanced maternal age and genetic predisposition, although the underlying pathogenesis remains incompletely unclear (Vince et al., 2020).

Fine particulate matter (PM_2.5_) is a major air pollutant that has been associated with adverse pregnancy outcomes, including stillbirth, preterm birth, low birth weight, and impaired fetal growth and development (Ghosh et al., 2021). PM_2.5_ exposure has been reported to be associated with insulin resistance and abnormal glucose metabolism potentially via oxidative stress and inflammatory responses (Liu et al., 2022). A growing body of epidemiological evidence has examined the association between PM_2.5_ exposure and GDM and generally suggests that higher PM_2.5_ exposure is associated with increased GDM risk, although heterogeneity remains across populations, exposure windows, and pollutant mixtures/components (Liang et al., 2023; Nazarpour et al., 2023; Chen et al., 2024). A recent prospective cohort study in Chinese women reported associations of gestational and postpartum exposure to PM_2.5_ components with glucose metabolism, highlighting the potential importance of PM_2.5_ composition and exposure windows (Chen et al., 2024). However, important gaps remain in determining the susceptible exposure windows and the potentially toxic components of PM_2.5_, as well as in clarifying the biological pathways that link PM_2.5_ exposure to dysglycemia and GDM.

Gut microbiota dysbiosis has been linked to T2DM and GDM, but the pathogenesis remains unclear, which may be related to the increase in the number of pathogenic bacteria and bile acid metabolism pathway (Kc et al., 2020). Although gut microbiota dysbiosis has been observed during pregnancy, research results are inconsistent, and no consistent GDM-specific signature has been identified (Huang et al., 2021). PM_2.5_ exposure has been associated with alterations in gut microbiota, which may relate to blood glucose homeostasis. The potential mechanism may be that PM_2.5_ is associated with inflammation and may increase intestinal permeability (Alderete et al., 2017). Evidence suggests that the gut microbiota may be involved in the associations between PM_2.5_ exposure and metabolic pathways such as sphingolipid and bile acid metabolism (Liu et al., 2019; Zhao et al., 2022). Our recent study evaluated the mediating role of gut microbiota in the association between PM_2.5_ exposure and GDM risk, reporting partial mediation by specific taxa (Long et al., 2025). However, human pregnancy studies that integrate specific PM_2.5_ exposure windows with gut microbiota and systemic molecular profiles to elucidate the underlying biological pathways remain relatively scarce.

Pregnant women with GDM exhibit alterations in blood metabolites (Bentley-Lewis et al., 2015). GDM-associated differential metabolites are mainly involved in metabolic pathways such as fatty acid metabolism, arachidonic acid metabolism, butyric acid metabolism, amino acid metabolism and bile secretion metabolism (Zhou et al., 2021). CircRNAs have been suggested as potential regulators of GDM (Zhang et al., 2021). However, the precise mechanisms underlying their role in GDM development remain unclear. CircRNAs may contribute to the pathogenesis of GDM by influencing insulin resistance and glycolipid metabolism (Chen et al., 2021). However, integrated epidemiological evidence linking PM_2.5_ exposure, gut microbiota, circulating metabolites, and circRNAs in relation to GDM is still scarce. Our prior study found that pregnant women with GDM exhibited gut microbiota dysbiosis, and identified differential metabolites and altered circRNAs expression (Mei et al., 2025).

In this study, we hypothesized that PM_2.5_ exposure is associated with changes in gut microbiota, blood metabolites and circRNAs, and that gut microbiota may modify the associations between PM_2.5_ exposure and glycemic outcomes, including GDM. First, we investigated the associations of residential PM_2.5_ exposure during different stages of pregnancy with the risk of GDM, blood glucose levels and gut microbiota composition. Second, we assessed whether the gut microbiota moderated the relationship between PM_2.5_ exposure and GDM. Lastly, we explored the interaction network among PM_2.5_ exposure, gut microbiota, blood metabolites and circRNAs as well as their associations with GDM, and explored related metabolic pathways, targets and potential mechanisms.

## Materials and methods

### Participants

Participants were selected from the Pregnancy Metabolic Disease and Adverse Pregnancy Outcomes (PMDAPO) study cohort, a dynamic prospective cohort of pregnant women established at Guangzhou Women and Children’s Medical Center. Details of the cohort have been reported previously (Yang et al., 2020; Gan et al., 2022). Pregnant women were included if they received antenatal care and delivered at Guangzhou Women and Children’s Medical Center. Pregnant women were excluded if they had pre-existing diabetes, pre-existing hypertension, pre-eclampsia, artificial insemination and *in-vitro* fertilization, psychiatric diseases, or resided outside of Guangzhou from three months before pregnancy until delivery.

**Figure 1:**
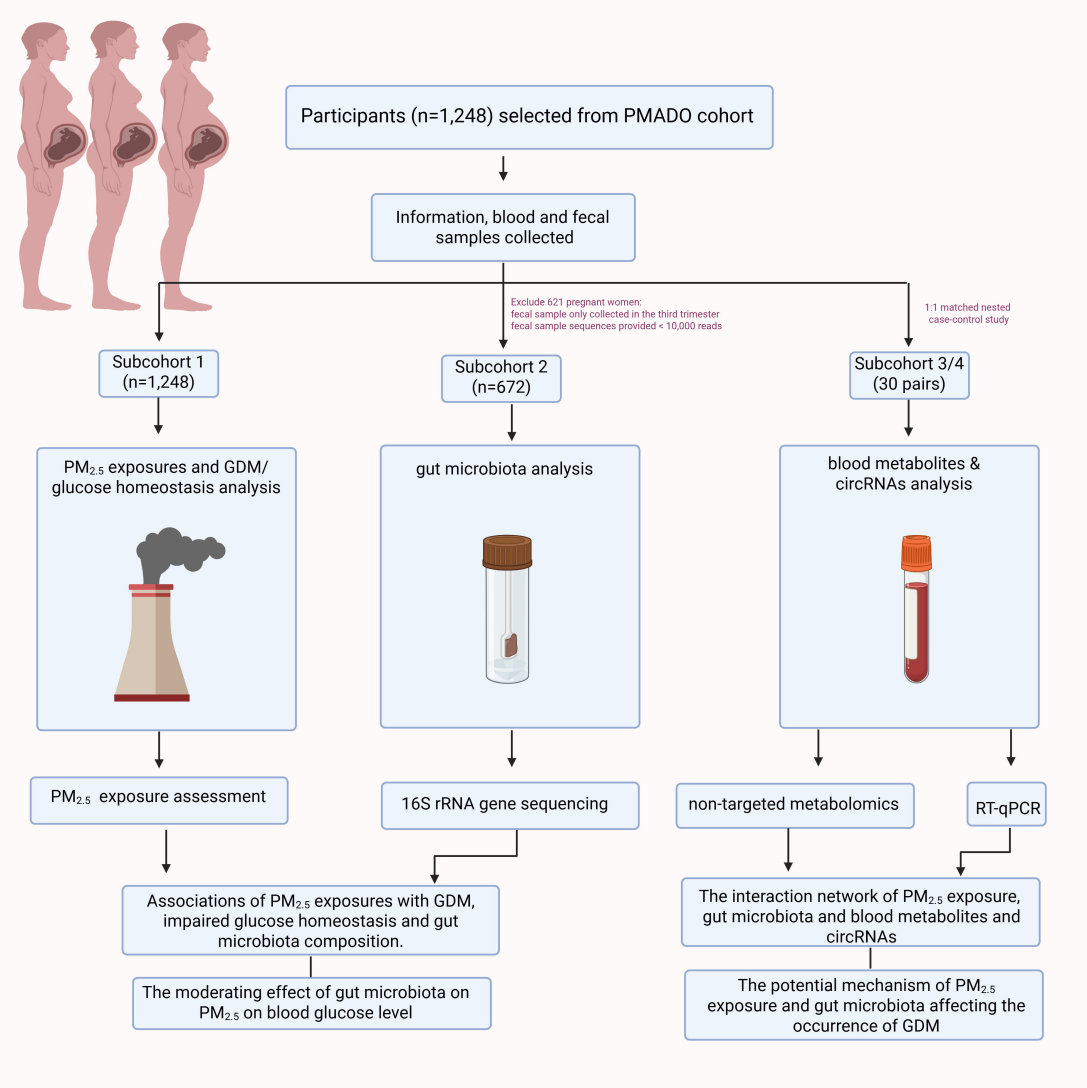
Schematic representation of the study. Created in BioRender. (https://BioRender.com/fla2etr).

A total of 1,248 pregnant women with available fecal samples were selected from the PMDAPO cohort between January 2017 and February 2020 (Subcohort 1) to investigate the associations between PM_2.5_ exposure, GDM, and impaired glucose homeostasis. After excluding 576 pregnant women whose fecal samples were collected only in the third trimester or fecal sample sequences provided < 10,000 reads, 672 pregnant women were included in the gut microbiota analysis (Subcohort 2). Using 1:1 matched nested case-control study, the GDM-associated blood metabolites (Subcohort 3) and circRNAs (Subcohort 4) were analyzed based on 30 pairs of pregnant women with GDM and matched controls without GDM, randomly selected from the PMDAPO cohort. The control subjects were matched with GDM women for maternal age (± 3 years), gestational weeks (± 3 weeks), gravidity (± 1) and parity (± 1). A schematic overview of the study is shown in **Figure 1**.

The protocol for this study was approved by the ethics review committee of Guangzhou Medical University, and all participants provided their voluntary signed informed consent.

### Clinical data collection and GDM assessment

At the first antenatal visit, information was collected on maternal age, gravidity, parity, last menstrual period, native place, residence address during pregnancy, pre-pregnancy weight, height, disease status, history of abortion, usage of antibiotics and other medication (including immunotoxic drugs and high-dose of commercial probiotics in the past six months or during pregnancy), conventional antibiotic treatment or probiotic supplementation in the preceding 4 weeks. Pre-pregnancy body mass index (BMI) was determined by calculating the pre-pregnancy weight divided by height squared (kg/m^2^). The season from 3 months before pregnancy to the 13th week of gestation was calculated based on the last menstrual period. Follow-up visits were performed according to routine obstetric examination intervals. Maternal clinical information was obtained from hospital medical records. The gestational age was determined by ultrasound examination.

A 75-g oral glucose tolerance test (OGTT) was conducted between 24 and 28 weeks of gestation. GDM was diagnosed when the following plasma glucose thresholds were met or exceeded at any point in time: fasting blood glucose of 5.1 mmol/L (92 mg/dL), 1-h blood glucose of 10.0 mmol/L (180 mg/dL), and 2-h blood glucose of 8.5 mmol/L (153 mg/dL) (American Diabetes Association Professional Practice Committee, 2022).

### Air pollutant exposure level assessment

Ambient air pollutant exposure for the participants was estimated based on the residential address reported at enrollment (first antenatal visit). Daily (24-h average) pollutant monitoring data on sulfur dioxide (SO_2_), nitrogen dioxide (NO_2_), and particulate matter < 2.5 μm in aerodynamic diameter (PM_2.5_) were collected from 11 National Air Quality Monitoring Stations in Guangzhou, China from the national urban air quality real-time publishing platform (http://106.37.208.233:20035/). The longitude and latitude (X1, Y1) of the pregnant women’s residential addresses and the longitude and latitude (X2, Y2) of each monitoring station were determined using online Coordinates Identification System provided by Baidu Map (http://api.map.baidu.com/lbsapi/getpoint/index.html) (Wu et al., 2018). The squared Euclidean distance was used to identify the nearest monitoring station, and the calculation formula was: D^2^=(X1−X2)^2^+(Y1−Y2)^2^. The air pollutant data from the monitoring station closest to each participant’s residence were used as their individual exposure level (Su et al., 2020). Based on the last menstrual period and delivery date of each participant, the average daily exposure concentration of each pollutant during 3 months before pregnancy, the first trimester (1-13 weeks), the second trimester (14-27 weeks), the third trimester (28 weeks to delivery), 3 months before pregnancy to the first trimester, and 3 months before pregnancy to the second trimester was calculated as the exposure level of each participant in each exposure window.

### Specimen collection and testing

Fecal samples were collected once per participant between 13 and 28 weeks of gestation, following a standardized and detailed protocol as previously described (Liu et al., 2019). The median gestational age at fecal sample collection was 19.3 weeks (IQR: 16.6-24.0). This window coincided with routine antenatal visits and partially overlapped with the standard OGTT screening window (24-28 weeks), which is etiologically relevant to GDM diagnosis and glucose homeostasis. In brief, pregnant women were instructed to follow standardized procedures prior to sample collection. All fecal samples were subpackaged and stored at -80°C within 30 minutes of collection. Total bacterial DNA was extracted from fecal samples using the MOBIO PowerSoil^®^ DNA Isolation Kit (12888-100) protocol and subsequently profiled through 16S rRNA gene (V4 region) sequencing. The V4 region of the 16S rRNA gene was amplified using universal primers 515F (5’-GTGYCAGCMGCCGCGGTAA-3’) and 806R (5’-GGACTACNVGGGTWTCTAAT-3’), with barcode sequences specific to each sample. The 16S rRNA amplicon sequences were processed using QIIME2 at Promegene Co. Ltd. (Shenzhen, China) in the same batch. Representative sequences were defined as merged sequences with 100% identity. The taxonomy of these representative sequences was identified using the classify-sklearn classification method based on the Greengenes 13.8 database (https://data.qiime2.org/2018.11/common/gg-13-8-99-515-806-nb-classifier.qza). The Greengenes database was selected to maintain methodological consistency with key prior work in this field, facilitating direct comparison and integration of findings (Mutlu et al., 1987; Ferrocino et al., 2018; Liu et al., 2019; Wei et al., 2022; Long et al., 2025).

Fasting blood samples were collected between 15 and 24 weeks of gestation. Serum was separated immediately for metabolite measurement and plasma was used for circRNA measurement. Based on our previous circRNA microarray profiling among pregnant women with GDM, the levels of the eight most relevant circRNAs were validated in 30 pairs of pregnant women (with and without GDM) by RT-qPCR. RT-qPCR was performed according to a standardized and detailed protocol (Yang et al., 2020).

Metabolomic analysis of the serum samples was conducted using untargeted liquid chromatography coupled with mass spectrometry (LC–MS; ACQUITY UPLC & Q-TOF Premier, Waters, Manchester, UK). An ACQUITY UPLC HSS T3 column (2.1 × 100 mm, 1.8 μm, Waters) was used in the LC–MS system, and the column oven was set to 40 °C. Solvent A consisted of water with 0.1% formic acid, while solvent B was acetonitrile with 0.1% formic acid. The flow rate was set to 0.3 mL/min, and the gradient elution was as follows: 0-2 min, 95% A;2-12 min, 95% A;12-15 min, 5% A;15-17 min, 5% A, 17-20 min, 95% A. Data acquisition was conducted in centroid mode using electrospray ionization (ESI) in both positive (ESI+) and negative (ESI−) modes over the mass-to-charge ratio (m/z) range of 50–1500, with a scan time of 0.2 s. For positive and negative modes, the capillary voltages were set to 1.4 kV and 1.3 kV, respectively, and the cone voltages to 40 V and 23 V, respectively. The source temperature was set to 120°C, with a cone gas flow of 50 L/h and a desolvation gas flow of 600 L/h. The desolvation temperature was set to 350°C and the collision energy was ramped from 10 to 40 V.

### Statistical analysis

Data are presented as terms of mean ± SD or n (%). Chi-square tests were used for categorical variables, and t tests for continuous variables. Spearman correlation analysis was conducted to assess correlations among exposure levels of PM_2.5_, SO_2_ and NO_2_. Multivariate logistic regression was employed to estimate the relative risk (RR) and 95% confidence intervals (95% CIs) of GDM associated with each 10 μg/m³ increase in PM_2.5_ exposure during specific exposure windows. Linear regression models were used to analyze the associations of PM_2.5_ exposure (increments of 10 μg/m³) on blood glucose levels during each exposure window, with fasting, 1-h and 2-h blood glucose levels as the dependent variables, respectively. In the single-pollutant model, independent variables included PM_2.5_ exposure concentration, age, season of pregnancy, gravidity, parity, and pre-pregnancy BMI. The multi-pollutant model additionally included SO_2_ and NO_2_ exposure concentrations, based on the single-pollutant model.

Spearman correlation analysis was used to analyze the correlation between PM_2.5_ exposure concentrations and gut microbiota. Hierarchical regression analysis was used to explore whether gut microbiota associated with PM_2.5_ exposure moderated the relationship between PM_2.5_ exposure and blood glucose levels. The genus significantly associated with PM_2.5_ exposure was used as the moderator variable M, PM_2.5_ exposure level as the independent variable X, and fasting, 1-h and 2-h blood glucose levels of OGTT as the dependent variables Y, respectively. All variables (X, M, and Y) were standardized. In the first step, the main effects of X and M on Y were examined using multiple linear regression, with X and M as independent variables, and Y as the dependent variable, adjusting for age, gravidity, parity, pre-pregnancy BMI, and exposure concentrations of SO_2_ and NO_2_. In the second step, the interaction term (X × M) was added to examine the moderating effect of gut microbiota on the association between PM_2.5_ exposure and blood glucose levels. The moderation model can be expressed as Y=β_0_+β_1_X+β_2_M+β_3_XM, where β_3_ quantifies effect modification. A positive β_3_ indicates a stronger PM_2.5_–glucose association at higher genus abundance, whereas a negative β_3_ indicates attenuation.

Metabolite data extraction and preprocessing were performed using MassLynx 4.1 mass spectrometry workstation software. Then, data normalization, principal component analysis (PCA), partial least squares discriminant analysis (PLS-DA) and orthogonal partial least squares discriminant analysis (OPLS-DA) were performed using SIMCA-P+ 13.0 (Umetrics, Umea, Sweden). Metabolite candidates were identified based on their mass-to-charge ratio (m/z) by searching for accurate masses in online databases, including the Human Metabolite Database (HMDB, v3.6), Kyoto Encyclopedia of Genes and Genomes (KEGG), and METLIN Metabolome Database. The metabolite candidates were selected based on the variable importance in projection (VIP) score combined with a two-sided t-test (VIP > 1, *P* < 0.05). High-throughput metabolic pathway enrichment analysis was performed using the MetaboAnalyst 5.0 platform, and metabolic network mapping, topological analysis and prediction of targets were performed using Cytoscape 3.9.1.

Based on the normal distribution of circRNA expression levels analyzed by the Kolmogorov-Smirnov test, the paired t-test or the Wilcoxon test was applied to evaluate differences in circRNA expression between participants with and without GDM. Spearman correlation analysis was used to analyze the associations among GDM-associated gut microbiota, PM_2.5_-related microbiota involved in blood glucose regulation, and GDM-associated metabolites and circRNAs.

All data were duplicated with Epidata3.1, and analyzed with R software (version 4.2.1). A two-sided test was used, with the significance level set at α = 0.05.

## Results

### Characteristics of the participants

Pregnant women in Subcohort 1 and Subcohort 2 were 18-45 years old, with GDM incidence of 19.15% (239/1,248) and 18.90% (127/672), respectively. Compared with those without GDM, pregnant women with GDM in both subcohorts were older and had higher pre-pregnancy BMI, gravidity, parity, fasting blood glucose, 1-h OGTT glucose, and 2-h OGTT glucose (*P* < 0.05). No statistically significant difference in abortion history was observed between women with and without GDM (*P* > 0.05) (**Table 1**). No statistically significant differences were found between the two subcohorts in terms of GDM incidence, age, pre-pregnancy BMI, gravidity or parity (**Supplementary Table S1**). For Subcohort 2, in which gut microbiota was analyzed, the median gestational age at fecal sample collection was 19.3 weeks (IQR: 16.6-24.0 weeks).

**Table 1:** Baseline characteristics in pregnant women from subcohort1 and subcohort2.

Characteristics	Category	Subcohort1 (N = 1248)		Subcohort2 (N = 672)	
		GDM n= (239, %)	Non-GDM (n=1009, %)	GDM (n=127, %)	Non-GDM (n=545, %)
Age (years)	X ± SD	32.48 ± 4.19	30.37 ± 4.20**	31.98 ± 4.18	30.17 ± 4.00**
Age group (years)	≤29	58 (24.27)	464 (45.99)	32 (25.20)	261 (47.89)
	30-34	99 (41.42)	384 (38.06)	60 (47.24)	206 (37.80)
	≥35	82 (34.31)	161 (15.96)	35 (27.56)	78 (14.31)
Pregnancy season	Spring	34 (14.23)	246 (24.38)**	27 (21.26)	138 (25.32)
	Summer	16 (6.69)	63 (6.24)	1 (0.79)	1 (0.18)
	Autumn	92 (38.49)	264 (26.16)	27 (21.26)	89 (16.33)
	Winter	97 (40.59)	436 (43.21)	72 (56.69)	317 (58.17)
History of abortion	Yes	79 (33.05)	311 (30.82)	40 (31.50)	159 (29.17)
	No	160 (66.95)	698 (69.18)	87 (68.50)	386 (70.83)
Gravidity (times)	1	73 (30.54)	412 (40.83)**	39 (30.71)	225 (41.28)*
	≥2	167 (69.46)	597 (59.17)	88 (69.29)	320 (58.72)
Parity (times)	0	106 (44.35)	559 (54.40)**	57 (44.88)	304 (55.78)*
	≥1	133 (55.65)	450 (44.60)	70 (55.12)	241 (44.22)
pre-pregnancy weigh (kg)	X ± SD	54.80 ± 7.48	52.14 ± 7.46**	54.44 ± 8.55	51.65 ± 7.02**
Pre-pregnancy BMI (kg/m^2^)	X ± SD	21.86 ± 2.82	20.54 ± 2.81**	21.68 ± 3.35	20.34 ± 2.50**
	<18.5	32 (13.39)	235 (23.29)**	19 (14.96)	138 (25.32)**
	18.5-23.9	154 (64.44)	666 (66.01)	81 (63.78)	358 (65.69)
	≥24	51 (21.34)	103 (10.21)	25 (19.69)	47 (8.62)
	Missing	2 (0.84)	5 (0.50)	2 (1.57)	2 (0.37)
Fasting Glucose (mmol/L)	X ± SD	4.64 ± 0.36	4.36 ± 0.36**	4.58 ± 0.41	4.32 ± 0.30**
1-hour Glucose of OGTT (mmol/L)	X ± SD	9.96 ± 1.70	7.35 ± 1.71**	9.93 ± 1.41	7.40 ± 1.32**
2-hour Glucose of OGTT (mmol/L)	X ± SD	9.32 ± 1.52	6.63 ± 1.51**	9.27 ± 1.23	6.57 ± 0.98**

*P < 0.05, **P < 0.01.

### Exposure levels of air pollutants and their correlation

**Supplementary Table S2** showed the exposure levels of air pollutants (including PM_2.5_, SO_2_ and NO_2_) in different exposure windows, including 3 months before pregnancy, the first trimester, the second trimester, the third trimester, from 3 months before pregnancy to the 13th week of gestation, and from 3 months before pregnancy to the 27th week of gestation. Spearman correlation analysis showed that the exposure levels of PM_2.5_, SO_2_ and NO_2_ were significantly positively correlated with each other in each exposure window (*P* < 0.05) (**Supplementary Table S3**).

### Associations of PM_2.5_ exposure with GDM and impaired glucose homeostasis

**Figure 2:**
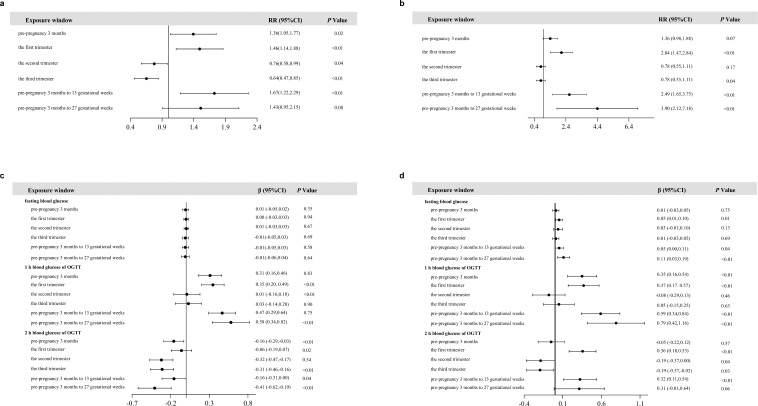
Associations of PM_2.5_ exposure with GDM and blood glucose. **(a)** Logistic regression analysis of the associations between PM_2.5_ exposure and GDM (single-pollutant model). **(b)** Logistic regression analysis of the associations between PM_2.5_ exposure and GDM (multi-pollutant model). **(c)** Linear regression analysis of the associations between PM_2.5_ exposure and blood glucose (single-pollutant model). **(d)** Linear regression analysis of PM_2.5_ exposure and blood glucose (multi-pollutant model).

The association of PM_2.5_ exposure with GDM and impaired glucose homeostasis was analyzed among 1,248 pregnant women (Subcohort 1). Results from the single-pollutant logistic regression model showed that, after adjusting for age, pre-pregnancy BMI, pregnancy season, gravidity and parity, the risk of GDM increased by 36% (RR = 1.36, 95% CI: 1.05, 1.77), 46% (RR = 1.46, 95% CI: 1.14, 1.88) and 67% (RR = 1.67, 95% CI: 1.22, 2.29) for every 10 μg/m^3^ increase in PM_2.5_ exposure during the three months before pregnancy, the first trimester, and from 3 months before pregnancy to the 13th week of gestation, respectively (**Figure 2a**). After further adjustment for SO_2_ and NO_2_, the results of the multi-pollutant model showed that the risk of GDM increased by 104% (RR = 2.04, 95% CI: 1.47, 2.84), 149% (RR = 2.49, 95% CI: 1.65, 3.75) and 290% (RR = 3.90, 95% CI: 2.12, 7.18) for every 10 μg/m^3^ increase in PM_2.5_ exposure during the first trimester, from 3 months to the 13th weeks of gestation, and from 3 months to the 27 weeks of gestation, respectively (**Figure 2b**).

The results of the single-pollutant linear regression model showed that for every 10 μg/m^3^ increase in PM_2.5_ exposure during the first trimester, the second trimester, and from 3 months before pregnancy to the 27th week of gestation, 1-h OGTT glucose increased by 0.35 mmol/L (95% CI: 0.20, 0.49), 0.01 mmol/L (95% CI: -0.16, 0.18), and 0.58 mmol/L (95% CI: 0.34, 0.82), respectively (**Figure 2c**). After adjustment for SO_2_ and NO_2_, the results of the multi-pollutant model showed that for every 10 μg/m^3^ increase in PM_2.5_ exposure during the first trimester, from 3 months before pregnancy to the 13th week of gestation, and from 3 months before pregnancy to the 27th week of gestation, fasting blood glucose increased by 0.05 mmol/L (95% CI: 0.01, 0.10), 0.05 mmol/L (95% CI: 0.00, 0.11) and 0.11 mmol/L (95% CI: 0.03, 0.19), respectively. For every 10 μg/m^3^ increase in PM_2.5_ exposure during the first trimester, the second trimester, from 3 months before pregnancy to the 13th week of gestation, and from 3 months before pregnancy to the 27th week of gestation, 1-h OGTT glucose increased by 0.35 mmol/L (95% CI: 0.16, 0.54), 0.37 mmol/L (95% CI: 0.17, 0.57), 0.59 mmol/L (95% CI: 0.34, 0.84) and 0.79 mmol/L (95% CI: 0.42, 1.16), respectively. For every 10 μg/m^3^ increase in PM_2.5_ exposure during the first trimester and from 3 months before pregnancy to the 13th week of gestation, 2-h OGTT glucose increased by 0.36 mmol/L (95% CI: 0.18, 0.53) and 0.32 mmol/L (95% CI: 0.11, 0.54), respectively (**Figure 2d**).

### Associations of diversity and composition of the gut microbiota with GDM and impaired glucose homeostasis

Based on 672 pregnant women (Subcohort 2), the results of the Wilcoxon test showed that there were no statistically significant differences in gut microbiota α-diversity (such as Simpson index, Shannon index, Chao1 index and Pielou_e index) between pregnant women with GDM and those without GDM (*P* > 0.05, **Supplementary Figure S1**). Based on the principal coordinates analysis (PCoA) of Bray-Curtis, the results showed that there were no significant differences in gut microbiota β-diversity between pregnant women with GDM and controls (**Supplementary Figure S2**).

**Figure 3:**
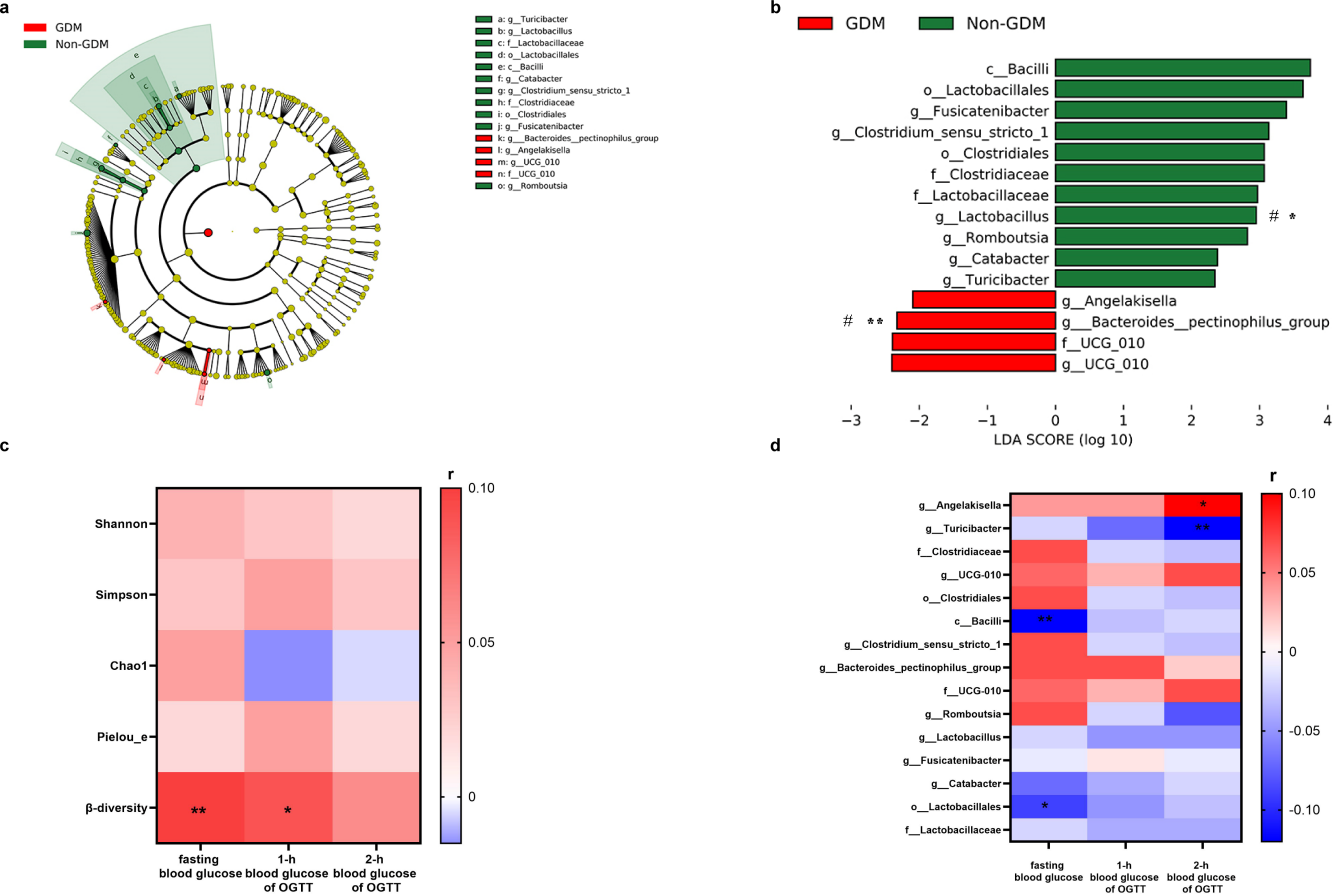
Differential gut microbiota between pregnant women with GDM and non-GDM (Subcohort2, n=672). **(a)** Cladogram of differential gut microbiota was identified by LEfSe analysis. **(b)** LDA scores of differential gut microbiota. Criteria: *P* < 0.05 and LDA score (log 10) ≥ 2.0. Blue bars indicate the bacterial taxa with greater relative abundance in the pregnant women without GDM; red bars indicate the gut microbiota with greater relative abundance in the GDM pregnant women. ^#^MaAsLin2, adjusted for age, pre-pregnancy BMI, gravidity and parity. **(c)** Spearman correlation analysis of OGTT blood glucose levels with α-diversity index and β-diversity index. **(d)** Spearman correlation analysis of OGTT blood glucose levels and GDM-associated differential bacteria. ^**^P < 0.01, ^*^P < 0.05.

The results of LEfSe analysis (LDA>2) showed that there were 15 differentially abundant taxa between pregnant women with GDM and those without GDM. At the class and order levels, Bacilli, Clostridiales and Lactobacillales were found to be enriched in non-GDM women. At the family level, Lactobacillales and Clostridiales were also enriched in non-GDM women, while the UCG-010 family was enriched in GDM women. At the genus level, *Turicibacter, Lactobacillus, Fusicatenibacter, Clostridium_sensu_stricto_1, Catabacter* and *Romboutsia* were enriched in non-GDM women, while *Bacteroides pectinophilus group, UCG-010* and *Angelakisella* were enriched in GDM women. After adjusting for age, pre-pregnancy BMI, gravidity and parity, the MaAsLin2 analysis showed that there were still significant differences in *Bacteroides pectinophilus group* and *Lactobacillus* between GDM and non-GDM women (**Figures 3a, b**).

The results of Spearman correlation analysis showed that there was no significant correlation between blood glucose levels and the α diversity indices of gut microbiota (*P* > 0.05). Fasting blood glucose and 1-h OGTT glucose were positively correlated with β- diversity of gut microbiota (*P* < 0.05; **Figure 3c**). The results of Spearman correlation analysis also showed that 37 bacteria were correlated with blood glucose levels. Among them, SCFAs-producing bacteria or inflammation-related bacteria *Eggerthella, Rothia*, *Streptococcus* and *Ruminococcus torques group* were negatively correlated with fasting blood glucose, while *Bacteroides*, *Colidextribacter*, *Lachnospiraceae UCG-008* and *Parabacteroides* were positively correlated with fasting blood glucose. *Anaerostipes* was positively correlated with 1-h OGTT glucose, whereas *Turicibacter* and *Odoribacter* were negatively correlated with 2-h OGTT glucose. After adjusting for age, pre-pregnancy BMI, gravidity and parity, *Eggerthella*, *Parabacteroides*, *Candidatus Stoquefichus*, *Ruminococcus torques group* and *Anaerostipes* were correlated with blood glucose levels (**Supplementary Table S4**).

Meanwhile, fasting blood glucose was negatively correlated with GDM-associated gut microbiota, including the class Bacilli and the order Lactobacillales, while 2-h OGTT glucose was negatively correlated with the genus *Turicibacter* and positively correlated with the genus *Angelakisella* (*P* < 0.05) (**Figure 3d**; **Supplementary Table S5**).

### The moderating effect of gut microbiota on the relationship between PM_2.5_ and blood glucose levels

The average daily exposure from 3 months before pregnancy to the 27th week of gestation was used to represent each pregnant woman’s exposure levels to air pollutants. Among 672 pregnant women (Subcohort 2), the average exposure concentrations of PM_2.5_, SO_2_ and NO_2_ were 36.54 μg/m^3^, 12.11 μg/m^3^ and 55.57 μg/m^3^, respectively (**Supplementary Table S6**). PM_2.5_ exposure levels were not significantly correlated with the Simpson index, Shannon index, Chao1 index or Pielou_e index of gut microbiota α diversity (*P* > 0.05), but significantly positively correlated with β diversity (r = 0.64, *P* < 0.01) (**Supplementary Table S7**).

**Figure 4:**
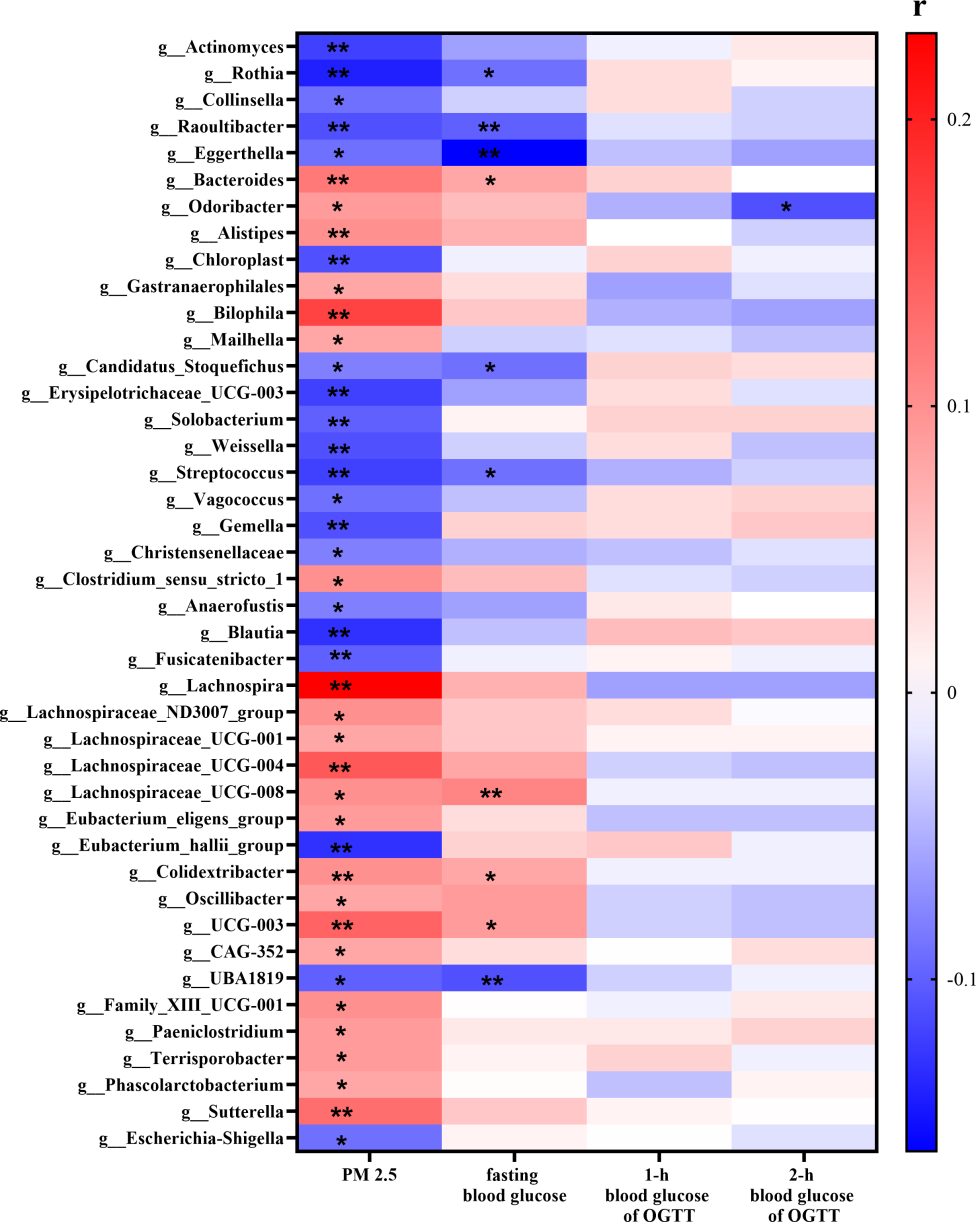
Spearman correlation analysis of PM_2.5_, blood glucose level and gut microbiota. ^**^P < 0.01, ^*^P < 0.05.

The results of Spearman correlation analysis showed that PM_2.5_ exposure levels were significantly correlated with 42 bacterial genera (*P* < 0.05). Among them, PM_2.5_ exposure levels were positively correlated with *Bacteroides, Odoribacter, Alistipes, Bilophila, Lachnospira, Sutterella, Terrisporobacter, Mailhella* and 14 other bacterial genera. Additionally, PM_2.5_ exposure levels were negatively correlated with 22 bacterial genera, such as *Rothia, Raoultibacter*, *Gemella*, *Solobacterium*, *Fusicatenibacter*, *Eubacterium hallii group* and *Escherichia-Shigella* (**Figure 4**; **Supplementary Table S8**).

Spearman correlation analysis between blood glucose levels and PM_2.5_-associated genera showed that fasting blood glucose was negatively correlated with *Rothia* (r = -0.09), *Raoultibacter* (r = -0.10), *Eggerthella* (r = -0.16), *Candidatus Stoquefichus* (r = -0.09), *Streptococcus* (r = -0.09) and *UBA1819* (r = -0.11), while it was positively correlated with *Bacteroides* (r = 0.08), *Lachnospiraceae_UCG-008* (r = 0.11), *Colidextribacter* (r = 0.08) and *UCG-003* (r = 0.09) (*P* < 0.05). Additionally, 2-h OGTT glucose was negatively correlated with *Odoribacter* (r = -0.11, *P* < 0.05) (**Figure 4**; **Supplementary Table S9**).

The moderating effects of PM_2.5_ exposure-related gut microbiota on blood glucose levels were explored using hierarchical regression analysis. To evaluate effect modification, we included an interaction term (PM_2.5_ × genus abundance) in the regression models. A statistically significant interaction indicates that the association between PM_2.5_ exposure and glucose levels varies across different levels of the gut microbial genus. The main effect was the effect of PM_2.5_ exposure (X) and gut microbiota (M) on blood glucose levels (Y) after controlling for age, pre-pregnancy BMI, gravidity, parity, SO_2_ and NO_2_. The interaction terms were the moderating effect of gut microbiota on the effects of PM_2.5_ exposure on blood glucose levels. The results showed that 7 bacterial genera significantly modified the association between PM_2.5_ exposure and blood glucose levels (*P* < 0.05; **Table 2**). For example, the positive association between PM_2.5_ exposure and fasting blood glucose was stronger among women with higher relative abundance of *Solobacterium* (β: 23.53, 95% CI: 4.33, 42.73, *P* = 0.02) and *Escherichia_Shigella* (β: 0.17, 95% CI: 0.00, 0.34, *P* = 0.04). In contrast, the association between PM_2.5_ and1-h OGTT glucose was attenuated among women with higher abundance of *Fusicatenibacter* (β: -1.10, 95% CI: -2.12, -0.07, *P* = 0.04) and *Ruminococcaceae_UBA1819* (β: -15.85, 95% CI: -29.91, -1.79, *P* = 0.03). Similarly, higher abundance of *Raoultibacter* (β: -267.20, 95% CI: -504.32, -30.08, *P* = 0.03)*, Anaerofustis* (β: -221.50, 95% CI: -411.87, -31.13, *P* = 0.02), *Ruminococcaceae_UBA1819* (β: -19.54, 95% CI: -31.80, -7.27, *P* < 0.01) and *Phascolarctobacterium* (β: -1.88, 95% CI: -3.11, -0.65, *P* < 0.01) attenuated the association between PM_2.5_ exposure and 2-h OGTT glucose.

**Table 2:** The moderating effect of gut microbiota on PM_2.5_ on blood glucose level.

Dependent variable	Moderator variable	Effect type	Model term	β	*95% CI* of β	*P*	ΔR^2^
Fasting glucose	g:Solobacterium	Main effect	X_1_	0.02	(0.01, 0.03)	<0.01	
			M_1_	-7.00	(-56.06, 42.06)	0.78	
		Interaction	X_1_×M_1_	23.53	(4.33, 42.73)	0.02	0.007^**^
	g:Escherichia_Shigella	Main effect	X_2_	0.02	(0.01,0.03)	<0.01	
			M_2_	0.19	(-0.60,0.98)	0.63	
		Interaction	X_2_×M_2_	0.17	(0.00, 0.34)	0.04	0.005^**^
1-h Glucose of OGTT	g:Fusicatenibacter	Main effect	X_3_	0.07	(0.01, 0.12)	0.02	
			M_3_	1.42	(-2.37, 5.21)	0.46	
		Interaction	X_3_×M_3_	-1.10	(-2.12, -0.07)	0.04	0.006^**^
	g:Ruminococcaceae_UBA1819	Main effect	X_4_	0.07	(0.01, 0.13)	0.01	
			M_4_	13.03	(-32.73, 58.79)	0.58	
		Interaction	X_4_×M_4_	-15.85	(-29.91, -1.79)	0.03	0.007^**^
2-h Glucose of OGTT	g:Raoultibacter	Main effect	X_5_	0.03	(-0.45, 0.08)	0.21	
			M_5_	-18.53	(-845.22, 808.16)	0.96	
		Interaction	X_5_×M_5_	-267.20	(-504.32, -30.08)	0.03	0.006^**^
	g:Anaerofustis	Main effect	X_6_	0.03	(-0.01,0.08)	0.17	
			M_6_	809.62	(80.59, 1538.64)	0.03	
		Interaction	X_6_×M_6_	-221.50	(-411.87, -31.13)	0.02	0.007^**^
	g:Ruminococcaceae_UBA1819	Main effect	X_7_	0.03	(-0.02, 0.08)	0.21	
			M_7_	1.68	(-38.47, 41.82)	0.93	
		Interaction	X_7_×M_7_	-19.54	(-31.80, -7.27)	<0.01	0.013^**^
	g:Phascolarctobacterium	Main effect	X_8_	0.03	(-0.02, 0.08)	0.20	
			M_8_	1.02	(-2.87, 4.91)	0.61	
		Interaction	X_8_×M_8_	-1.88	(-3.11, -0.65)	<0.01	0.012^**^

M: Moderator variable. It is the bacteria genus significantly associated with PM_2.5_ exposure.

X: Independent variable. It is the PM_2.5_ exposure level.

X×M: The interaction term between the bacteria genus significantly associated with PM_2.5_ exposure and the PM_2.5_ exposure level. A positive coefficient for X×M indicates that the PM_2.5_–glucose association is stronger at higher genus abundance, whereas a negative coefficient indicates attenuation.

ΔR^2^: Change of the adjusted coefficient of determination.

**P < 0.01.

### Functional analysis of GDM-associated differential blood metabolites and circRNAs

**Figure 5:**
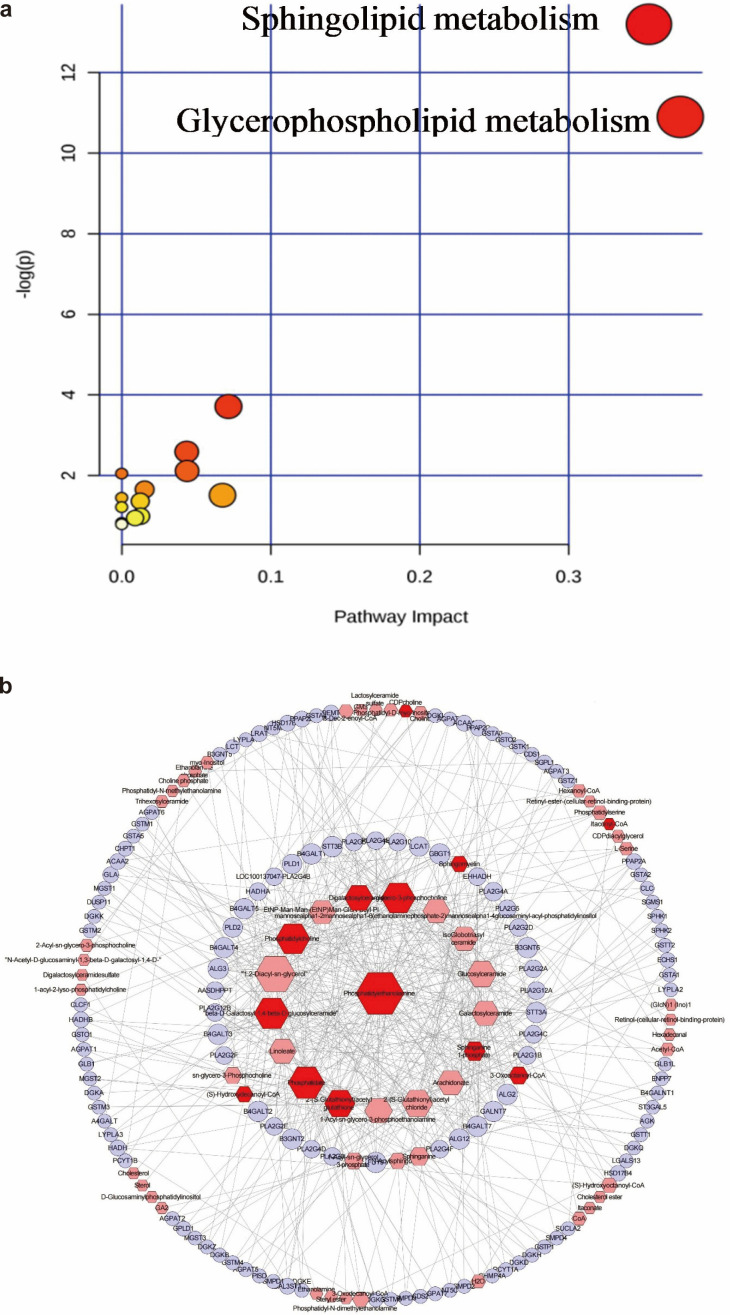
Analysis of different metabolites between GDM pregnant women and controls. **(a)** Pathway enrichment analysis of GDM-associated differential blood metabolites. The larger the impact value of the horizontal coordinate pathway, the larger the bubble, and the greater the role of metabolites in the pathway. The larger the ordinate-log (P) value, the redder the color, and the more significant the metabolic pathway. **(b)** Metabolomic network diagram of GDM-associated differential blood metabolites. The red hexagon represents the GDM-associated differential metabolites; the pink hexagon represents compounds related to GDM-associated differential metabolites; purple circles represent genes associated with GDM-associated differential metabolites. The node degree value of the central node is the largest, and the node degree value of the compounds and genes of its adjacent two circles is greater than the average node degree.

Univariate significance tests were performed to compare all the metabolites, identifying significant differences between pregnant women with GDM and those without GDM. A total of 28 GDM-associated differential blood metabolites were identified (**Supplementary Table S10**). High-throughput metabolic pathway enrichment analysis based on these 28 differential metabolites of GDM showed that glycerophospholipid metabolism and sphingolipid metabolism may be involved in GDM (**Figure 5a**; **Supplementary Table S11**).

Topological data analysis showed that 11 GDM-associated differential metabolites had node degrees higher than the average, including phosphatidylethanolamine (degree = 39), Phosphatidic acid (degree = 26), Dolichyl β-D-glucosyl phosphate (degree = 25), Lysophosphatidylcholine (degree = 24), Phosphatidylcholine (degree = 24), Galactosylceramide (degree = 18), sphinganine-1-phosphate (degree = 10), 2-(S-Glutathionyl)acetyl glutathione (degree = 20), 3-Oxooctanoyl-CoA (degree = 8), Sphingomyelin (degree = 6) and (S)-Hydroxydecanoyl-CoA (degree = 6). The 11 differential metabolites were involved in 9 metabolic pathways, including glycerophospholipid metabolic, Glycosphingolipid metabolism, Phosphatidylinositol phosphate metabolism, Linoleate metabolism, Glycosphingolipid biosynthesis-globo series, Glycosphingolipid biosynthesis-ganglio series, Arachidonic acid metabolism, Saturated fatty acids β-oxidation and Xenobiotics metabolism, respectively (**Figure 5b**; **Supplementary Table S12**). Based on topological data analysis, the target genes of the GDM-associated differential metabolites were predicted using key node centrality indicators, including degree centrality (DC), betweenness centrality (BC) and closeness centrality (CC). Genes with DC, BC and CC values above the mean were selected as potential targets of GDM-associated differential metabolites (**Supplementary Table S13**). *PLD1*, *PLD2*, *EHHADH* and *HADHA* were selected as key target genes. *PLD1* and *PLD2* were involved in the glycerophospholipid and phosphatidylinositol phosphate metabolic pathways, while *EHHADH* and *HADHA* were involved in the saturated fatty acid β-oxidation pathway.

RT-qPCR analysis showed that several circRNAs, including hsa_circ_0001946, hsa_circ_0000154, hsa_circ_0006732, hsa_circ_0001016 and hsa_circ_0001439 were upregulated in GDM, while hsa_circ_0042852, hsa_circ_0004001 and hsa_circ_0006936 were downregulated in GDM (**Supplementary Table S14**). Signaling pathway prediction based on KEGG database showed that hsa_circ_0006732 and hsa_circ_0001439 were related to the insulin signaling pathway and the biological process of insulin response.

**Figure 6:**
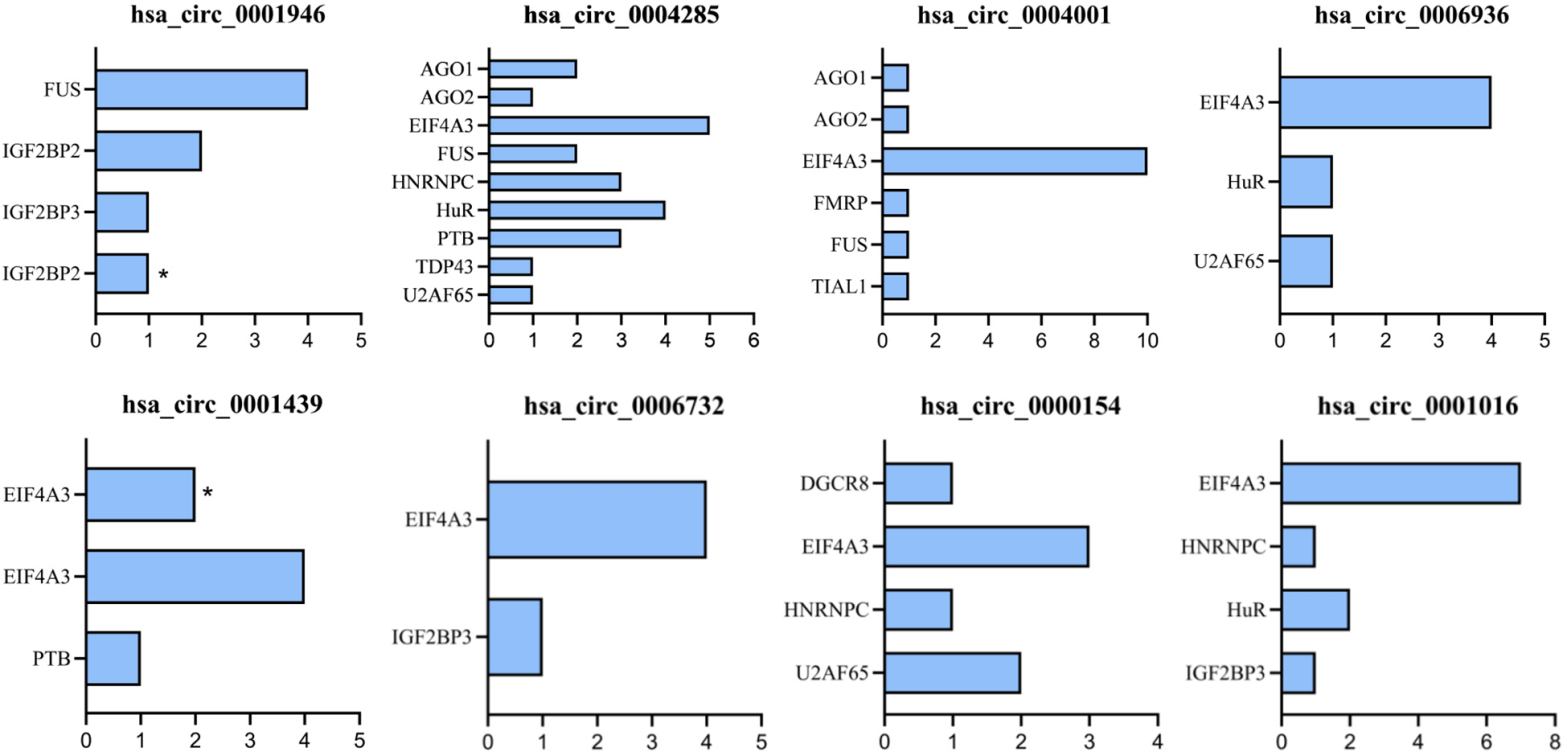
RNA-binding proteins (RBPs) and the number of predicted binding sites (count) in the flanking regions or back-splice junction (linker) region of GDM-associated circRNAs (predicted by CircInteractome). Bars indicate the number of predicted RBP binding sites (count). *indicates RBPs with predicted binding sites in the linker/back-splice junction region.

circRNA binding proteins were predicted by the CircInteractome database. The results showed that hsa_circ_0001946 had four kinds of binding proteins in the lateral region, among which IGF2BP2 had binding sites in both the lateral region and junction region. hsa_circ_0001439 had two kinds of lateral region-binding proteins, among which EIF4A3 had binding sites in both lateral region and junction region. EIF4A3 was identified as a common binding site for hsa_circ_0042852, hsa_circ_0004001, hsa_circ_0006936, hsa_circ_0001439, hsa_circ_0006732, hsa_circ_0000154, and hsa_circ_0001016. IGF2BP3 served as a shared binding protein for hsa_circ_0001946, hsa_circ_0006732 and hsa_circ_0001016 (**Figure 6**). Both IGF2BP2 and IGF2BP3 were RNA-binding proteins that interact with insulin-like growth factors. AGO proteins were involved in the processing and maturation of small RNAs, while EIF4A3 protein was associated with translation regulation.

### Association of GDM-associated gut microbiota, GDM-associated metabolites and circRNAs

**Figure 7:**
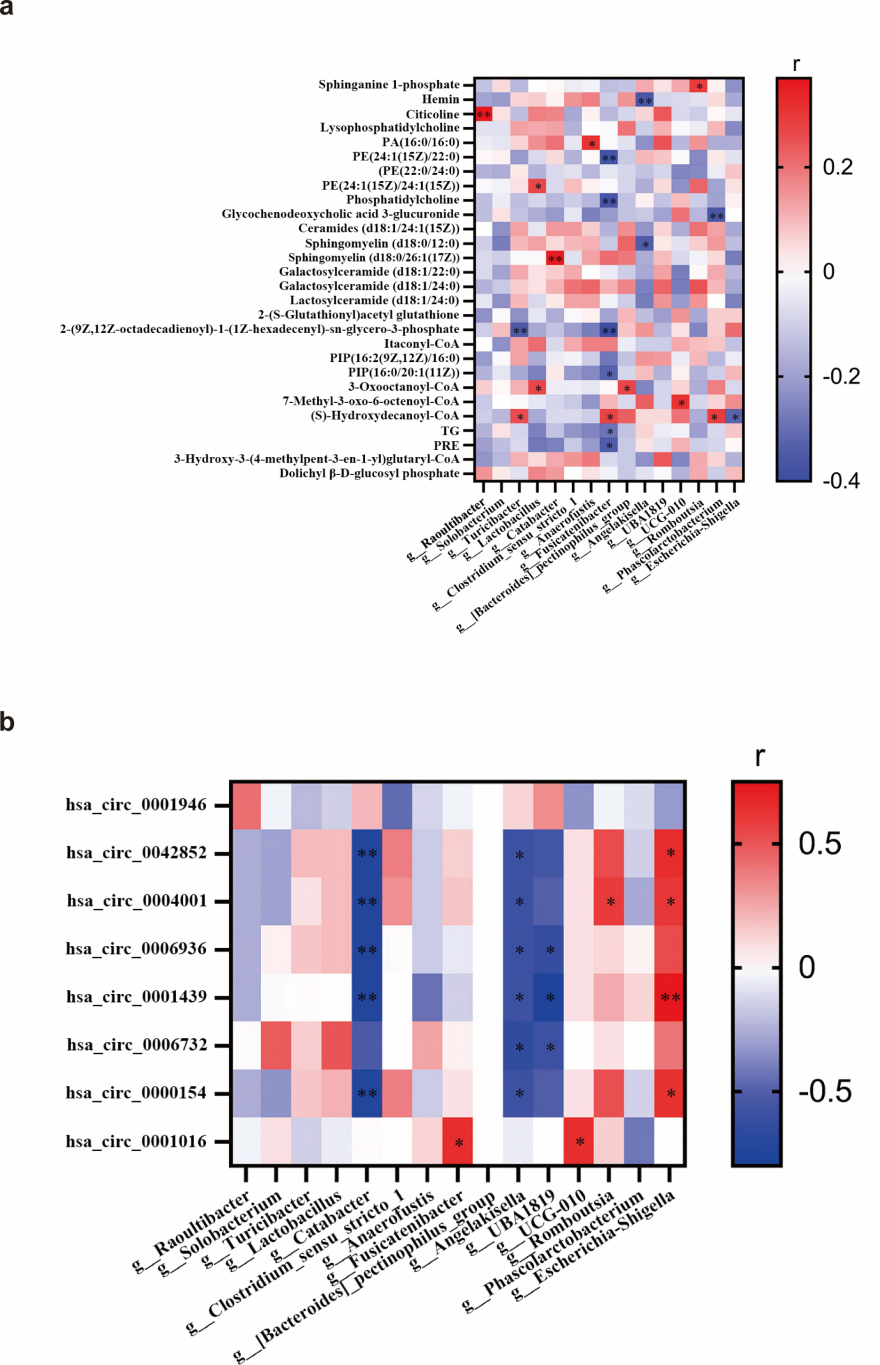
Association of GDM-associated gut microbiota and GDM-associated metabolites and circRNAs. **(a)** Spearman correlation analysis of GDM-associated gut microbiota and GDM-associated differential metabolites. **(b)** Spearman correlation analysis of GDM-associated gut microbiota and GDM-associated differential circRNA. ^**^P < 0.01, ^*^P < 0.05.

Spearman correlation analysis showed that 12 GDM-associated gut microbial genera were significantly correlated with 17 GDM-associated differential metabolites. Among them, the GDM-associated differential metabolites sphinganine-1-phosphate and Sphingomyelin (d18:0/26:1(17Z)), annotated to the sphingolipid metabolic pathway, were positively correlated with *Romboutsia* and *Catabacter*, respectively. Sphingomyelin (d18:0/12:0) was negatively correlated with *Angelakisella*, which was enriched in pregnant women with GDM. Among the GDM-associated differential metabolites involved in the glycerophospholipid metabolic pathway, PE (24:1(15Z)/24:1(15Z)) was positively correlated with *Lactobacillus*, which was enriched in non-GDM pregnant women. Citicoline and PA (16:0/16:0) were positively correlated with *Raoultibacter* and *Anaerofustis*, respectively; both genera showed negative effect modification of the association between PM_2.5_ exposure and 2-h OGTT glucose. PE (24:1(15Z)/22:0) and PC (24:0/24:1(15Z)) were negatively correlated with *Fusicatenibacter*, which was also enriched in non-GDM pregnant women and inhibited the association between PM_2.5_ exposure and 1-h OGTT glucose (**Figure 7a**; **Supplementary Table S15**).

Spearman correlation analysis showed that seven GDM-associated bacterial genera were correlated with seven GDM-associated circRNAs. Among them, hsa_circ_0042852 and hsa_circ_0000154 were negatively correlated with *Catabacter*, enriched in non-GDM pregnant women, and with *Angelakisella*, enriched in GDM pregnant women. Conversely, both circRNAs were positively correlated with *Escherichia-Shigella*, which was associated with a stronger association between PM_2.5_ and fasting blood glucose. hsa_circ_0004001 was negatively correlated with *Catabacter* and *Angelakisella*, but was positively correlated with *Romboutsia*, enriched in non-GDM pregnant women, and with *Escherichia-Shigella*. hsa_circ_0006936 was negatively correlated with *Catabacter*, *Angelakisella*, and *Ruminococcaceae*_*UBA1819*, the latter of which showed negative effect modification of PM_2.5_ on 2-h OGTT glucose. hsa_circ_0001439 was negatively correlated with *Catabacter*, *Angelakisella* and *Ruminococcaceae_UBA1819*, but positively correlated with *Escherichia-Shigella*. hsa_circ_0006732 was negatively correlated with *Angelakisella* and *Ruminococcaceae_UBA1819*. hsa_circ_0001016 was positively correlated with *Oscillospirales_UCG-010*, enriched in GDM pregnant women, and with *Fusicatenibacter*, enriched non-GDM pregnant women, which showed negative effect modification of PM_2.5_ on 1-h OGTT glucose (**Figure 7b**; **Supplementary Table S16**).

## Discussion

Our study found that higher PM_2.5_ exposure was associated with a higher risk of GDM and higher blood glucose levels. The findings showed that gut microbiota could moderate the association between PM_2.5_ and blood glucose levels. Specifically, *Solobacterium* and *Escherichia_Shigella* had a positive effect modification on the association between PM_2.5_ and fasting blood glucose, while *Fusicatenibacter*, *Ruminococcaceae_UBA1819*, *Raoultibacter*, *Anaerofustis* and *Phascolarctobacterium* had a negative effect modification on the association between PM_2.5_ exposure and 2-h OGTT glucose. Because fasting/1-h/2-h OGTT glucose are the diagnostic components of GDM, effect modification observed for these glycemic traits may indicate differential susceptibility to PM_2.5_-related dysglycemia and could be relevant to GDM risk. The findings also showed that gut microbiota which were associated with GDM or could moderate the effect of PM_2.5_ on blood glucose levels, were significantly associated with GDM-associated metabolites (such as sphinganine-1-phosphate, Sphingomyelin, phosphatidylcholine (PC), phosphatidic acid (PA) and Citicoline) and GDM-associated circRNAs (such as hsa_circ_0006732 and hsa_circ_0001439). Moreover, *Catabacter* and *Angelakisella*, which were enriched in pregnant women with GDM, and *Romboutsia* and *Fusicatenibacter*, enriched in those without GDM, were associated with both GDM-associated metabolites and GDM-associated circRNAs. The results of high-throughput metabolic pathway enrichment analysis showed that GDM-associated differential metabolites related to gut microbiota were enriched in two pathways: glycerophospholipid and sphingolipid metabolic pathways. In contrast, GDM-associated differentially expressed circRNAs were linked to the insulin signaling pathway and participated in the biological process of insulin response. These findings highlight the potential roles of PM_2.5_ exposure and gut microbiota dysbiosis in the development of GDM.

Our results showed that elevated PM_2.5_ exposure was associated with an increased risk of GDM and higher maternal blood glucose levels of OGTT. The period from 3 months before pregnancy to the 27th week of gestation might be a susceptible window during which PM_2.5_ exposure is more strongly associated with GDM. These results were consistent with previous studies (Lin et al., 2020). Notably, inverse associations were observed between PM_2.5_ exposure during the second and/or third trimesters with GDM risk and 2-h OGTT glucose (**Figure 2**). These counterintuitive findings should be interpreted cautiously and do not necessarily indicate a protective effect of PM_2.5_. GDM was ascertained by a 75-g OGTT at 24–28 gestational weeks, whereas the third-trimester exposure window was defined as 28 weeks to delivery (American Diabetes Association Professional Practice Committee, 2022). Thus, third-trimester exposure is largely post-ascertainment and is not etiologically informative for GDM onset or OGTT measurements. In addition, trimester-specific PM_2.5_ averages are strongly influenced by seasonal patterns and time-varying factors, and residual confounding or instability across correlated exposure windows may contribute to inverse estimates in later pregnancy. And defining late-pregnancy exposure as 28 weeks to delivery may introduce bias related to delivery timing. Therefore, we emphasize preconception/early pregnancy and cumulative exposure up to 27 weeks as the more etiologically relevant windows, while the inverse associations in later windows warrant confirmation in future studies. Studies have shown that long-term exposure to air pollution can increase the endogenous stress response in pregnant women, resulting in excessive secretion of hormones such as insulin, adrenaline and cortisol, which will increase the blood glucose levels of pregnant women (Kodavanti, 2016; Lin et al., 2020). Abnormal fasting blood glucose levels were associated with increased insulin resistance and reduced basal insulin secretion, while abnormal postprandial blood glucose levels were associated with impaired β-cell function and mild insulin resistance (Ma AG and Ra, 2006). The observed association between PM_2.5_ exposure and GDM may be related to insulin resistance and pancreatic islet β-cell dysfunction. However, the mechanisms underlying the relationship between PM_2.5_ exposure and the development of GDM remain unclear. Studies have shown that PM_2.5_ can lead to insulin resistance through the inflammatory response, oxidative stress and endothelial cell function impairment (Haberzettl et al., 2016).

In our study, no significant differences were observed in the α and β diversity of gut microbiota between pregnant women with GDM and those without GDM from the 13th to the 28th week of gestation. However, fasting blood glucose and 1-h OGTT glucose were positively correlated with the β diversity of the gut microbiota. These findings suggested that there was no significant difference in gut microbiota diversity between pregnant women with GDM and those without GDM, while blood glucose levels may be associated with the β diversity of gut microbiota. This was consistent with previous studies (Hasan et al., 2018; Wei et al., 2022).

The results of this study showed that the gut microbiota in pregnant women with GDM was dysregulated. After adjusting for age, gravidity, parity and pre-pregnancy BMI, the abundance of *Bacteroides_pectinophilus_group* remained increased in pregnant women with GDM, while *Lactobacillus* increased in pregnant women without GDM. Fasting glucose levels were negatively correlated with *Eggerthella, Candidatus Stoquefichus and Ruminococcus torques group*, and positively correlated with *Parabacteroides*. 1-h OGTT glucose levels were positively correlated with *Anaerostipes*. *Bacteroides pectinophilus group* belongs to the family Lachnospiraceae, which is a Gram-negative bacterium producing Lipopolysaccharides (LPS). Studies have shown that Lachnospiraceae is enriched in pregnant women with GDM and is associated with blood glucose levels, suggesting that it may play a key role in glucose metabolism (Wang et al., 2020). *Lactobacillus* is a beneficial genus of bacterium that has been reported to be associated with lower LPS levels and reduced inflammation, and may improve insulin sensitivity, thereby potentially reducing the risk of GDM (Zhang et al., 2021).

*Parabacteroides*, which is positively associated with elevated blood glucose levels, is one of the core bacterial genera in the human microbiome. Studies have shown that *Parabacteroides* is negatively correlated with obesity and diabetes, suggesting it may play a beneficial regulatory role in inflammation, glucose and lipid metabolism (Cui et al., 2022). However, another study showed that *Parabacteroides* was enriched in pregnant women with GDM and might be a characteristic feature of their gut microbiome (Kuang et al., 2017). These findings suggest that *Parabacteroides* may be involved in GDM and possibly other types of diabetes. *Parabacteroides* can hydrolyze various conjugated bile acids and convert them into secondary bile acids, including lithocholic acid, ursodeoxycholic acid (Wang et al., 2019; Liu et al., 2023). Secondary bile acids such as lithocholic acid may improve lipid metabolism disorders by activating the intestinal FXR signaling pathway. Ursodeoxycholic acid can help repair the integrity of intestinal wall (Fiorucci et al., 2018; Golden et al., 2018). Succinic acid, the main metabolite of *Parabacteroides*, can activate intestinal gluconeogenesis by acting on fructose-1, 6-bisphosphatase (FBPase), a key enzyme in the intestinal gluconeogenesis (IGN) pathway. It also promotes hepatic glycogen synthesis, and may improve glucose metabolism in the host (Wang et al., 2019).

*Anaerostipes*, which was positively correlated with 1-h OGTT glucose, may be associated with inflammation. A study showed that the levels of thiamine and indoleacrylic acid, which were related to inflammation, were significantly positively correlated with *Anaerostipes* (Zhong et al., 2024). Another study showed that *Anaerostipes* was involved in inositol metabolism. Some strains of *Anaerostipes* spp. metabolized inositol to produce short-chain fatty acids (SCFAs), such as propionic acid and butyric acid under anaerobic conditions *in vitro* (Bui et al., 2021). Inositol metabolism-related gene clusters were negatively correlated with metabolic markers in the diabetic population.

*Eggerthella*, *Candidatus Stoquefichus* and *Ruminococcus torques group*, which were negatively correlated with fasting blood glucose levels, may be involved in the regulation of lipid metabolism and inflammation (Pinart et al., 2022). Studies have shown that serum TNF-α and IL-4 are negatively correlated with *Candidatus Stoquefichus*, suggesting its potential anti-inflammatory role. A reduced abundance of this bacterium may lead to increased expression of inflammatory mediators (Yang et al., 2021). *Ruminococcus torques group* belongs to *Ruminococcus*, which can ferment resistant starch, dietary fiber and oligosaccharides to produce short-chain fatty acids such as butyrate. Butyrate helps maintain intestinal barrier function, supports microbial balance, regulate immunity and anti-inflammation (Wang et al., 2020; Xiao et al., 2020). These findings suggest that gut microbiota may be involved in the occurrence of GDM by inducing inflammatory responses, thereby affecting SCFAs production and insulin resistance.

Long-term exposure to PM_2.5_ has been associated with gut microbiota dysbiosis (Mutlu et al., 1987). This study also found that 42 genera were associated with PM_2.5_ exposure, of which 11 genera were associated with fasting blood glucose and/or 2-h OGTT glucose. *Rothia*, *Raoultibacter*, *Eggerthella*, *Candidatus Stoquefichus*, *Streptococcus* and *Ruminococcaceae UBA1819* were negatively correlated with both PM_2.5_ exposure and fasting blood glucose levels. Additionally, *Odoribacter* was negatively correlated with 2-h OGTT glucose. In contrast, *Bacteroides, Lachnospiraceae UCG-008*, *Colidextribacter* and *UCG-003* were positively correlated with both PM_2.5_ exposure and fasting blood glucose levels. *Rothia* and *Streptococcus* are SCFA-producing bacteria that produce butyric acid and acetic acid, respectively; both metabolites were negatively correlated with blood glucose levels. These bacteria were enriched in non-GDM women and may be linked to better glucose homeostasis (Rungrueang et al., 2021).

*Raoultibacter* has been reported to regulate the expression of bile acid synthases and reduce the risk of diabetes (Sato et al., 2021). *Eggerthella* is an opportunistic pathogen associated with a variety of chronic human diseases, including obesity and diabetes (Pinart et al., 2022; Jiang S et al., 2021). However, another study showed that the levels of *Eggerthella* were significantly higher at baseline before impaired glucose tolerance progressed to diabetes mellitus (Zhang et al., 2024), which was consistent with our findings. The association of *Candidatus Stoquefichus* and *Odoribacter* with GDM has not been previously reported. In a mouse model, β-carotene upregulated the relative abundance of *Candidatus Stoquefichus*, which had a negative linear correlation with the concentrations of both TNF-α and IL-4 (Yang X. et al., 2021). This suggested that *Candidatus Stoquefichus* may be linked to the development of GDM through inflammatory response. In a mouse model of high-fat diet (HFD) induced obesity, prebiotic treatment with oligofructose significantly increased the relative and absolute abundance of the genus *Odoribacter*, which was negatively associated with metabolic parameters such as body weight, fat mass and glucose metabolism (Paone et al., 2022). The proposed mechanisms may include modulation of gut microbiota, restoration of intestinal barrier function, alleviation of dysbiosis, reduction of chronic low-grade inflammation and regulation of gut peptide secretion (Vallianou et al., 2023).

Previous studies have shown that GDM women have a lower abundance of *Bacteroides* (Kamińska et al., 2022). However, other studies have also shown that different *Bacteroides* strains have different effects. A review of 10 observational studies on obesity reported higher levels of *Bacteroides fragilis* in obese individuals compared to non-obese individuals. In contrast, a review on diabetes showed lower levels of *Bacteroides vulgatus* (Michels et al., 2022). In this study, only 16S rRNA sequencing was used to identify bacterial genera. In the next step, metagenomic sequencing will be used to identify the dominant *Bacteroides* species and strains in GDM women.

*Lachnospiraceae UCG-008* has been reported to be positively correlated with inflammatory markers (such as IL-6, hs-CRP and TNF-α) and serum fasting insulin (Zhu et al., 2020). However, a previous study reported that treatment of HFD-induced obese mice with astilbin, which was known for its significant anti-inflammatory activity, could reduce insulin resistance and inflammation, accompanied by a decrease in the abundance of *Lachnospiraceae UCG-008* (Wang et al., 2022). *Colidextribacter* and *UCG-003*, both belonging to the Oscillospiraceae family, have been reported to produce short-chain fatty acids and contribute to glucose homeostasis. The abundance of Oscillospiraceae was associated with decreased insulin resistance, potentially due to their ability to produce short-chain fatty acids and support glucose homeostasis (Palmnäs-Bédard et al., 2022). However, Li et al. reported that the abundance of *Colidextribacter* was positively correlated with BMI and total cholesterol (Li et al., 2022). The inconsistency in the findings of different studies might be attributed to species and strain specific differences.

The results of the moderating effect analysis in our study showed that gut microbiota significantly modified the associations of PM_2.5_ exposure with blood glucose levels. Specifically, S*olobacterium* and *Escherichia_Shigella* showed positive effect modification on the association between PM_2.5_ and fasting blood glucose, while *Fusicatenibacter*, *Ruminococcaceae_UBA1819*, *Raoultibacter*, *Anaerofustis* and *Phascolarctobacterium* showed negative effect modification of the association between PM_2.5_ and 2-h OGTT glucose. *Solobacterium* and *Escherichia_Shigella* have been reported to be associated with pro-inflammatory features. *Solobacterium moorei* could activate the NF-κB signaling pathway, leading to chronic low-grade inflammation and destruction of the intestinal barrier (Yu et al., 2024). *Escherichia_Shigella*, an opportunistic pathogen belonging to the family Enterobacteriaceae, could increase intestinal epithelial permeability, induce macrophage apoptosis, and release IL-1β to induce intestinal inflammation (Sansonetti et al., 1999). *Fusicatenibacter*, *Ruminococcaceae_UBA1819*, *Anaerofustis* and *Phascolarctobacterium* are short-chain fatty acid (SCFA)-producing bacteria (Medawar et al., 2021; Yang et al., 2021). Moreover, *Phascolarctobacterium* and *Ruminococcaceae* were involved in secondary bile acid (SBA) biosynthesis (Yang et al., 2021). SCFAs and SBAs are the main gut microbiota metabolic products that may modulate inflammation and metabolic disorders (Medawar et al., 2021). Mechanistically, pro-inflammatory genera may increase intestinal permeability and systemic inflammatory signaling, thereby strengthening PM_2.5_-related metabolic stress and insulin resistance (Di Vincenzo et al., 2024; Mostafavi Abdolmaleky and Zhou, 2024), whereas SCFA/SBA-producing genera may improve barrier integrity and dampen inflammation/oxidative stress, attenuating the downstream disturbance of glucose homeostasis (Visekruna and Luu, 2021; Portincasa et al., 2022). In addition, emerging multi-omics evidence suggests that PM_2.5_ exposure can also reshape microbial metabolic networks, including lipid, amino-acid, and energy metabolism, which may further alter host insulin signaling and pregnancy metabolism through changes in SCFAs and bile acid pools (de Vos et al., 2022). The role of *Raoultibacter* has not been elucidated yet. A study reported that *Raoultibacter* was significantly positively correlated with body weight, blood lipids, and blood glucose-related indicators in mice with high-fat diet-induced obesity (Miao et al., 2023). Further research is required to understand the relationship among *Raoultibacter*, PM2.5 and blood glucose. The results of our study suggested that PM_2.5_ exposure might increase the abundance of pro-inflammatory bacteria or decrease the abundance of SCFAs producing bacteria, which is consistent with a potential role of inflammation and insulin resistance in the observed associations with GDM.

Our findings also showed that gut microbiota associated with GDM or capable of moderating the effect of PM_2.5_ on blood glucose levels were significantly associated with GDM-associated metabolites and circRNAs. Among the bacteria that attenuated the association between PM_2.5_ exposure and blood glucose levels, *Fusicatenibacter* was negatively correlated with phosphatidylethanolamine (PE), phosphatidylcholine (PC) and triglycerides (TG), which were involved in glycerophospholipid metabolism; *Phascolarctobacterium* was negatively correlated with glycochenodeoxycholic acid 3-glucuronide, involved in pentose and glucuronate interconversions, and was positively correlated with (S)-hydroxydecanoyl-CoA, involved in fatty acid metabolism. *Ruminococcaceae UBA1819*, which attenuated the association between PM_2.5_ on 2-h OGTT glucose, was negatively correlated with hsa_circ_0001439 and hsa_circ_0006732, both of which were involved in the insulin signaling pathway. *Escherichia-Shigella*, which showed positive effect modification of the PM_2.5_ association with fasting glucose, was positively correlated with hsa_circ_0001439 and negatively correlated with (S)-hydroxydecanoyl-CoA. These cross-omics correlations may reflect an interconnected pathway linking PM_2.5_ exposure, gut microbiota, and host metabolic responses, which may help explain the observed effect-modified associations between PM_2.5_ and glycemic traits. One plausible explanation is that certain microbes can reshape PM_2.5_-related metabolic perturbations by altering microbial and host lipid metabolism and energy utilization, thereby changing the downstream impact on insulin signaling (Zhang et al., 2023). This interpretation is supported by our pathway/network results showing that GDM differential metabolites clustered in glycerophospholipid metabolism and sphingolipid metabolism, and by metabolomics network topology analysis (consistent with MetaboAnalyst 5.0) highlighting glycerophospholipid metabolism. The key target genes *PLD1* and *PLD2* of the differential metabolites-pathway related gene network of GDM were involved in the glycerophospholipid and phosphatidylinositol phosphate metabolic pathway, and the other two key target genes *EHHADH* and *HADHA* were involved in the saturated fatty acid β-oxidation pathway. *PLD1* and *PLD2* are isozymes of phospholipase D, which play important roles in vesicle transport, phagocytosis, metabolic regulation and cytoskeleton organization. *PLD1* and *PLD2* are activated by cell surface receptors and hydrolyze phosphatidylcholine to produce phosphatidic acid (Hwang et al., 2021). Kim et al. reported that inhibition of *PLD2* expression could improve oral glucose tolerance and insulin sensitivity in mice (Kim et al., 2022). *EHHADH* is a bifunctional enzyme in the fatty acid β-oxidation pathway, which is negatively correlated with fasting blood glucose (Houten et al., 2012). Mitochondrial β-oxidase *HADHA* is considered a negative regulator of hepatic gluconeogenesis. Its overexpression can stimulate hepatic gluconeogenesis and destroy lipid metabolism (Pan et al., 2022). In addition, our bioinformatics analysis showed that EIF4A3 and IGF2BP3 were the flanking region binding proteins of hsa_circ_0001439 and hsa_circ_0006732, respectively. IGF2BP3 is an insulin-like growth factor binding protein, and EIF4A3 is associated with translational regulation. Anderlova et al. found that GDM was associated with IGFBP3 (Anderlová et al., 2022). In summary, our results suggest that PM_2.5_ and gut microbiota might influence the development of GDM through inflammation, glycerophospholipid metabolism, and the insulin signaling pathway, and that the potential targets may be *PLD1* and *PLD2* genes, as well as IGF2BP3 and EIF4A3 proteins.

### Advantages and limitations

This study has several advantages. To our knowledge, this is the first study that provided epidemiological evidence of the moderating effect of PM_2.5_ exposure, gut microbiota, plasma metabolites, circRNAs and GDM. The covariates could be adjusted with the large sample size. Based on the prospective cohort study, we were able to examine the longitudinal associations among PM_2.5_ exposure, gut microbiota, plasma metabolites, circRNAs and GDM.

However, several limitations of this study should be acknowledged. Due to the lack of work address information, PM_2.5_ exposure level was evaluated based solely on the participants’ residential addresses. And we relied on a single baseline address for exposure assessment and did not capture intra-city residential moves during pregnancy. This could result in non-differential misclassification of exposures with high spatial variability (e.g., air pollution). Future research would benefit from tracking address changes to enhance accuracy. In addition, detailed data on dietary intake and physical activity were not collected, and residual confounding by these factors cannot be ruled out. However, several factors mitigate the potential for confounding by these variables. All participants received standardized antenatal care at the Guangzhou Women and Children’s Medical Centre, which includes routine health education on balanced diet and appropriate physical activity. And their residential addresses during pregnancy were within Guangzhou, meaning they were exposed to similar regional dietary influences (traditional Cantonese diets are characteristically light and balanced), which may reduce extreme variation in dietary patterns. Besides, BMI was included as a covariate in all regression models, which was a strong proxy indicator of long-term energy balance influenced by both diet and physical activity. Third, the gut microbiota was assessed only once during mid-pregnancy. Although this period is key for GDM development, a single sample cannot reflect changes across early or late pregnancy. Therefore, our results provide a snapshot, and microbial or exposure effects at other times might also matter. It should be noted that our previous analysis found that the influence of gestational age on women’s gut microbiota composition was limited (Yang et al., 2020). Future studies featuring longitudinal tracking with multiple samples are required to establish precise temporal links. Fourth, this study did not evaluate interactions between PM_2.5_ and other air pollutants (e.g. PM_10_, CO, O_3_), nor did it account for meteorological conditions such as temperature and humidity. The fecal and blood specimens were collected from different pregnant women in the same cohort, which may have introduced some potential impact on the study results. However, the pregnant women were included in the cohort at the same period using the same inclusion and exclusion criteria, and the study results still offer valuable reference for future research. Fifth, this study utilized the Greengenes (v13.8) database for taxonomic annotation. While this database has been widely used in comparable studies to ensure consistency, we acknowledge that it is not the most recent version. Future studies employing updated databases such as SILVA for validation would be a valuable addition. Sixth, gut microbiota profiling was based on 16S rRNA gene (V4) amplicon sequencing, which has inherent limitations in taxonomic resolution (typically up to the genus level) and cannot reliably distinguish species- or strain-level variation. Moreover, 16S amplicon data are compositional and do not directly quantify microbial functional genes or pathways. Thus, functional interpretation should be made with caution. Future studies using shotgun metagenomic sequencing along with updated reference databases and longitudinal sampling would help validate species/strain-level signals and elucidate functional mechanisms.

## Conclusions

Our results supported a significant association between PM_2.5_ exposure and GDM risk/maternal blood glucose levels, and suggested that gut microbiota may moderate the effect of PM_2.5_ exposure on blood glucose levels. Our results suggested that PM_2.5_ and gut microbiota might be involved in the development of GDM through inflammation, glycerophospholipid metabolism and the insulin signaling pathway. The key targets may be *PLD1* and *PLD2* genes, as well as IGF2BP3 and EIF4A3 proteins.

## Data Availability

The datasets presented in this study can be found in online repositories. The names of the repository/repositories and accession number(s) can be found in the article/**Supplementary Material**.
