# Supplementary material for: Longitudinal associations between PM2.5 with gestational diabetes mellitus mediated by gut microbiome and potential mechanism: based on a prospective pregnant women cohort in China
Source: Front Cell Infect Microbiol. 2026 Feb 27;16:1749504. doi: 10.3389/fcimb.2026.1749504 (PMC12982432; doi:10.3389/fcimb.2026.1749504)
Supplement: Supplementary file 1 [file Table1.doc]

**Supplementary tables**

**Table S1** General Characteristics between pregnant women of subcohort1 and subcohort2

| General Characteristics |  | Subcohort1  N=1248 (%) | Subcohort2  N=672(%) | *P* |
| --- | --- | --- | --- | --- |
| Gestational diabetes mellitus | Yes | 1009(80.85) | 555(82.59) | 0.35 |
|  | No | 239(19.15) | 117(17.41) |
| Age (years) | $\overline{X}$± SD | 30.77±4.20 | 30.51±4.10 | 0.19 |
| Age group (years) | ≤29 | 522(41.83) | 293(45.29) | 0.35 |
|  | 30-34 | 483(38.70) | 266(41.11) |
|  | ≥35 | 243(19.47) | 113(17.46) |
| Gravidity (times) | 0 | 485(38.86) | 264(39.29) | 0.86 |
|  | ≥1 | 763(61.14) | 408(60.71) |
| Parity (times) | 0 | 665(53.28) | 361(53.72) | 0.85 |
|  | ≥1 | 583(46.71) | 311(46.28) |
| Pregnancy season | Spring | 280(22.44) | 165(24.55) | <0.01 |
|  | Summer | 79(6.33) | 2(0.30) |
|  | Autumn | 356(28.52) | 116(17.26) |
|  | Winter | 533(42.71) | 389(57.89) |
| History of abortion | Yes | 390 | 199 | 0.46 |
|  | No | 858 | 473 |
| Height (cm) | $\overline{X}$± SD | 159.15±5.21 | 159.14±5.20 | 0.96 |
| Weight (kg) | $\overline{X}$± SD | 52.65±7.47 | 52.17±7.40 | 0.18 |
| Pre-pregnancy BMI (kg/m^2^) | $\overline{X}$±SD | 20.79±2.81 | 20.59±2.73 | 0.14 |
|  | <18.50 | 267(21.39) | 157(23.36) | 0.41 |
|  | 18.50-23.99 | 820(65.71) | 439(65.33) |
|  | ≥24.00 | 154(12.34) | 72(10.71) |
|  | Missing | 7(0.56) | 4(0.60) |
| Fasting Glucose (mmol/L) | $\overline{X}$± SD | 4.41±0.36 | 4.37±0.34 | <0.01 |
| 1-hour Glucose (mmol/L) | $\overline{X}$± SD | 7.85±1.71 | 7.88±1.66 | 0.73 |
| 2-hour Glucose (mmol/L) | $\overline{X}$± SD | 7.14±1.51 | 7.08±1.48 | 0.35 |

**Table S2** The air pollutants (including PM_2.5_, SO_2_ and NO_2_) exposure levels in different exposure windows in subcohort1

|  | Min | P_25_ | ± SD | Median | P_75_ | Max | IQR |
| --- | --- | --- | --- | --- | --- | --- | --- |
| **3 months before pregnancy** | | | | | | | |
| SO_2_（μg/m^3^） | 3.72 | 10.40 | 11.86±2.91 | 12.48 | 13.62 | 24.50 | 3.22 |
| NO_2_（μg/m^3^） | 18.62 | 39.66 | 51.02±14.68 | 50.53 | 63.91 | 83.57 | 24.25 |
| PM_2.5_（μg/m^3^） | 16.91 | 30.79 | 37.80±8.31 | 36.81 | 44.28 | 54.90 | 13.49 |
| **First trimester (1-12 weeks of gestation)** | | | | | | | |
| SO_2_（μg/m^3^） | 3.59 | 10.30 | 11.35±2.75 | 12.20 | 12.95 | 17.34 | 2.65 |
| NO_2_（μg/m^3^） | 18.62 | 47.17 | 55.87±12.61 | 57.67 | 66.03 | 82.36 | 18.86 |
| PM_2.5_（μg/m^3^） | 15.86 | 32.11 | 37.77±9.43 | 38.98 | 44.91 | 54.38 | 12.80 |
| **Second trimester (13-24 weeks of gestation)** | | | | | | | |
| SO_2_（μg/m^3^） | 3.67 | 10.10 | 11.10±2.76 | 11.08 | 12.74 | 17.38 | 2.64 |
| NO_2_（μg/m^3^） | 17.07 | 44.27 | 51.70±12.02 | 50.75 | 60.69 | 83.12 | 16.42 |
| PM_2.5_（μg/m^3^） | 15.91 | 24.60 | 31.71±9.36 | 29.03 | 37.76 | 57.00 | 13.16 |
| **Third trimester (25-42 weeks of gestation)** | | | | | | | |
| SO_2_（μg/m^3^） | 3.72 | 9.79 | 11.10±2.66 | 11.15 | 12.15 | 18.06 | 2.36 |
| NO_2_（μg/m^3^） | 16.76 | 43.19 | 48.48±10.29 | 46.73 | 54.19 | 87.99 | 11.00 |
| PM_2.5_（μg/m^3^） | 13.79 | 24.58 | 30.62±8.07 | 29.27 | 35.20 | 63.55 | 10.62 |
| **From 3 months before pregnancy to 13th week of gestation** | | | | | | | |
| SO_2_（μg/m^3^） | 4.22 | 10.78 | 11.59±2.56 | 12.42 | 13.09 | 19.68 | 2.31 |
| NO_2_（μg/m^3^） | 19.59 | 46.16 | 53.53±11.91 | 54.13 | 62.98 | 78.88 | 16.82 |
| PM_2.5_（μg/m^3^） | 16.38 | 33.17 | 37.78±6.82 | 38.02 | 44.18 | 49.39 | 11.01 |
| **From 3 months before pregnancy to 27th week of gestation** | | | | | | | |
| SO_2_（μg/m^3^） | 4.30 | 10.75 | 11.42±2.47 | 12.03 | 12.80 | 16.99 | 2.05 |
| NO_2_（μg/m^3^） | 21.27 | 48.17 | 52.88±9.54 | 54.24 | 60.24 | 70.92 | 12.07 |
| PM_2.5_（μg/m^3^） | 21.51 | 32.62 | 35.68±4.71 | 36.69 | 39.38 | 44.00 | 6.76 |

Min, minimum value; P_25_, lower quartile; P_75_, upper quartile; Max, maximum value; IQR, interquartile range.

**Table S3** Spearman [correlation](javascript:;) [analysis](javascript:;) of air pollutants in different exposure windows in subcohort1

|  | PM_2.5_ | SO_2_ | NO_2_ |
| --- | --- | --- | --- |
| **3 months before pregnancy** | | | |
| PM_2.5_ | 1.00 | 0.31^**^ | 0.76^**^ |
| SO_2_ |  | 1.00 | 0.23^**^ |
| NO_2_ |  |  | 1.00 |
| **First trimester (1-12 weeks of gestation)** | | | |
| PM_2.5_ | 1.00 | 0.63^**^ | 0.66^**^ |
| SO_2_ |  | 1.00 | 0.53^**^ |
| NO_2_ |  |  | 1.00 |
| **Second trimester (13-24 weeks of gestation)** | | | |
| PM_2.5_ | 1.00 | 0.40^**^ | 0.71^**^ |
| SO_2_ |  | 1.00 | 0.50^**^ |
| NO_2_ |  |  | 1.00 |
| **Third trimester (25-42 weeks of gestation)** | | | |
| PM_2.5_ | 1.00 | 0.20^**^ | 0.62^**^ |
| SO_2_ |  | 1.00 | 0.39^**^ |
| NO_2_ |  |  | 1.00 |
| **From 3 months before pregnancy to 13th week of gestation** | | | |
| PM_2.5_ | 1.00 | 0.62^**^ | 0.64^**^ |
| SO_2_ |  | 1.00 | 0.42^**^ |
| NO_2_ |  |  | 1.00 |
| **From 3 months before pregnancy to 17th week of gestation** | | | |
| PM_2.5_ | 1.00 | 0.75^**^ | 0.50^**^ |
| SO_2_ |  | 1.00 | 0.50^**^ |
| NO_2_ |  |  | 1.00 |

^**^*P*＜0.01

**Table S4** Bacteria significantly associated with blood glucose levels

| Blood glucose index | Bacteria genus | r | *P* |
| --- | --- | --- | --- |
| Fasting glucose | Rothia | -0.09 | 0.02 |
|  | Raoultibacter | -0.10 | <0.01 |
|  | Eggerthella | -0.16 | <0.01 |
|  | Bacteroides | 0.08 | 0.04 |
|  | Butyricimonas | 0.08 | 0.03 |
|  | Paraprevotella | 0.08 | 0.03 |
|  | Parabacteroides | 0.10 | <0.01 |
|  | Candidatus_Stoquefichus | -0.09 | 0.02 |
|  | Coprobacillus | -0.11 | <0.01 |
|  | Faecalicoccus | -0.10 | <0.01 |
|  | Clostridium_innocuum_group | -0.09 | 0.02 |
|  | Streptococcus | -0.09 | 0.02 |
|  | Lachnospiraceae_GCA-900066575 | 0.11 | <0.01 |
|  | Lachnospiraceae_UCG-008 | 0.11 | <0.01 |
|  | Lactonifactor | -0.08 | 0.04 |
|  | Oribacterium | 0.08 | 0.04 |
|  | Eubacterium_fissicatena_group | -0.09 | 0.02 |
|  | Eubacterium_ruminantium_group | 0.11 | <0.01 |
|  | Ruminococcus_torques_group | -0.09 | 0.02 |
|  | Colidextribacter | 0.08 | 0.04 |
|  | Oscillospiraceae_UCG-003 | 0.09 | 0.02 |
|  | Oscillospiraceae_UCG-005 | 0.08 | 0.04 |
|  | Negativibacillus | 0.12 | <0.01 |
|  | Ruminococcaceae_UBA1819 | -0.11 | <0.01 |
|  | Peptococcus | 0.08 | 0.04 |
|  | Eubacterium_nodatum_group | -0.11 | <0.01 |
|  | Synergistes | 0.09 | 0.02 |
| 1-hour Glucose of OGTT | f__Prevotellaceae;g__uncultured | -0.10 | 0.01 |
|  | Faecalicoccus | -0.10 | 0.01 |
|  | Faecalitalea | 0.09 | 0.03 |
|  | Lactococcus | 0.08 | 0.04 |
|  | Anaerosporobacter | -0.13 | <0.01 |
|  | Anaerostipes | 0.08 | 0.04 |
|  | Tuzzerella | -0.10 | 0.01 |
| 2-hour Glucose of OGTT | Methanosphaera | 0.08 | 0.04 |
|  | Odoribacter | -0.09 | 0.02 |
|  | Turicibacter | -0.12 | <0.01 |
|  | Anaerosporobacter | -0.08 | 0.04 |
|  | Tuzzerella | -0.08 | 0.03 |
|  | Angelakisella | 0.10 | 0.01 |
|  | Negativibacillus | 0.08 | 0.03 |

**Table S5** The correlation between GDM-associatedgut microbiota with blood glucose levels **in subcohort1**

| Blood glucose index | GDM-associated bacteria | r | *P* |
| --- | --- | --- | --- |
| Fasting glucose | c__Bacilli | -0.11 | <0.01 |
| Fasting glucose | o__Lactobacillales | -0.09 | 0.02 |
| 2-h blood glucose of OGTT | g__Angelakisella | 0.10 | 0.01 |
| 2-h blood glucose of OGTT | g__Turicibacter | -0.12 | <0.01 |

**Table S6** The air pollutants (including PM_2.5_, SO_2_ and NO_2_) exposure levels from 3 months before pregnancy to the 27th week of gestation in subcohort2

|  | Min | P_25_ | X±SD | Median | P_75_ | Max | IQR |
| --- | --- | --- | --- | --- | --- | --- | --- |
| SO_2_（μg/m^3^） | 4.70 | 11.61 | 12.11±1.73 | 12.04 | 12.70 | 16.78 | 1.09 |
| NO_2_（μg/m^3^） | 21.27 | 52.97 | 55.57±8.88 | 57.39 | 61.49 | 70.92 | 8.52 |
| PM_2.5_（μg/m^3^） | 24.33 | 34.22 | 36.54±3.57 | 36.93 | 39.11 | 43.38 | 4.89 |

**Table S7** Spearman correlation analysis of PM2.5 exposure levels with α-diversity index and β-diversity index of gut microbiota in subcohort2

| Index |  | r | *P* |
| --- | --- | --- | --- |
| α-diversity | Simpson | 0.01 | 0.87 |
|  | Shannon | -0.01 | 0.74 |
|  | Chao1 | 0.05 | 0.23 |
|  | Pielou_e | -0.01 | 0.72 |
| β-diversity |  | 0.64 | <0.01 |

**Table S8** Spearman correlation analysis of PM2.5 exposure levels with gut microbiota in subcohort2

| Bacterial genera | r | *P* |  | Bacterial genera | r | *P* |
| --- | --- | --- | --- | --- | --- | --- |
| Actinomyces | -0.12 | <0.01 |  | Anaerofustis | -0.08 | 0.04 |
| Rothia | -0.14 | <0.01 |  | Blautia | -0.13 | <0.01 |
| Collinsella | -0.09 | 0.02 |  | Fusicatenibacter | -0.10 | <0.01 |
| Raoultibacter | -0.11 | <0.01 |  | Lachnospira | 0.23 | <0.01 |
| Eggerthella | -0.09 | 0.02 |  | Lachnospiraceae_ND3007_group | 0.10 | 0.01 |
| Bacteroides | 0.12 | <0.01 |  | Lachnospiraceae_UCG_001 | 0.08 | 0.04 |
| Odoribacter | 0.09 | 0.02 |  | Lachnospiraceae_UCG_004 | 0.15 | <0.01 |
| Alistipes | 0.10 | <0.01 |  | Lachnospiraceae_UCG_008 | 0.10 | 0.01 |
| Chloroplast | -0.11 | <0.01 |  | Eubacterium_eligens_group | 0.09 | 0.02 |
| Gastranaerophilales | 0.08 | 0.04 |  | Eubacterium_hallii_group | -0.13 | <0.01 |
| Bilophila | 0.17 | <0.01 |  | Colidextribacter | 0.10 | <0.01 |
| Mailhella | 0.08 | 0.03 |  | Oscillibacter | 0.08 | 0.04 |
| Candidatus_Stoquefichus | -0.08 | 0.03 |  | Oscillospiraceae_UCG-003 | 0.14 | <0.01 |
| Erysipelotrichaceae_UCG_003 | -0.12 | <0.01 |  | Ruminococcaceae_CAG-352 | 0.08 | 0.04 |
| Solobacterium | -0.10 | <0.01 |  | Ruminococcaceae_UBA1819 | -0.10 | 0.01 |
| Weissella | -0.11 | <0.01 |  | Family_XIII_UCG_001 | 0.10 | 0.01 |
| Streptococcus | -0.12 | <0.01 |  | Paeniclostridium | 0.09 | 0.02 |
| Vagococcus | -0.09 | 0.02 |  | Terrisporobacter | 0.09 | 0.02 |
| Gemella | -0.11 | <0.01 |  | Phascolarctobacterium | 0.08 | 0.04 |
| Christensenellaceae | -0.08 | 0.04 |  | Sutterella | 0.13 | <0.01 |
| Clostridium_sensu_stricto_1 | 0.10 | 0.01 |  | Escherichia_Shigella | -0.09 | 0.02 |

**Table S9** Spearman correlation analysis of blood glucose levels with gut microbiota in subcohort2

| Blood glucose | Bacterial genera | r | *P* |
| --- | --- | --- | --- |
| Fasting glucose | Rothia | -0.09 | 0.02 |
|  | Raoultibacter | -0.10 | <0.01 |
|  | Eggerthella | -0.16 | <0.01 |
|  | Bacteroides | 0.08 | 0.04 |
|  | Candidatus_Stoquefichus | -0.09 | 0.02 |
|  | Streptococcus | -0.09 | 0.02 |
|  | Lachnospiraceae_UCG-008 | 0.11 | <0.01 |
|  | Colidextribacter | 0.08 | 0.04 |
|  | Oscillospiraceae_UCG-003 | 0.09 | 0.02 |
|  | Ruminococcaceae_UBA1819 | -0.11 | <0.01 |
| 2-hour Glucose of OGTT | Odoribacter | -0.11 | 0.02 |

**Table S10** Differential metabolites between GDM pregnant women and controls

| NO | name | RT (min) | m/z | VIP | *P* | FC | HMDB | KEGG |
| --- | --- | --- | --- | --- | --- | --- | --- | --- |
| 1 | Phosphatidyl ethanolamine((PE(22:0/24:0)) | 14.04 | 888.71 | 1.41 | 0.04 | 21.27 | HMDB09508 | C00350 |
| 2 | Piperidine(16:0/20:1(11Z)) | 5.91 | 945.52 | 1.43 | 0.03 | 19.19 | HMDB34301 | C01746 |
| 3 | Phosphatidic acid(PA(16:0/16:0)) | 10.44 | 649.47 | 1.32 | 0.05 | 16.96 | HMDB00674 | C00416 |
| 4 | Phosphatidyl ethanolamine (PE(24:1(15Z)/22:0)) | 14.05 | 886.70 | 1.55 | 0.02 | 12.42 | HMDB09765 | C00350 |
| 5 | Piperidine(16:2(9Z,12Z)/16:0) | 14.05 | 887.51 | 1.54 | 0.02 | 9.49 | HMDB34301 | C01746 |
| 6 | Sphingomyelin (d18:0/26:1(17Z)) | 13.50 | 841.68 | 1.45 | 0.03 | 7.50 | HMDB01348 | C00550 |
| 7 | 2-(9Z,12Z-octadecadienoyl)-1-(1Z-hexadecenyl)-sn-glycero-3-phosphate | 11.03 | 657.48 | 1.68 | 0.01 | 6.77 | HMDB11155 | C15647 |
| 8 | Glycochenodeoxycholic acid 3-glucuronide | 10.74 | 626.40 | 1.44 | 0.03 | 6.64 | HMDB02579 | C03033 |
| 9 | Sphinganine 1-phosphate | 5.11 | 382.32 | 1.63 | 0.01 | 6.46 | HMDB01383 | C01120 |
| 10 | (S)-Hydroxydecanoyl-CoA | 5.90 | 938.30 | 1.28 | 0.04 | 5.18 | HMDB03938 | C05264 |
| 11 | Galactosylceramide (d18:1/22:0) | 15.23 | 946.73 | 1.36 | 0.03 | 4.36 | HMDB04836 | C06126 |
| 12 | 2-(S-Glutathionyl)acetyl glutathione | 10.58 | 655.15 | 1.21 | 0.04 | 3.80 | HMDB60343 | C14863 |
| 13 | 3-Hydroxy-3-(4-methylpent-3-en-1-yl)glutaryl-CoA | 15.35 | 980.23 | 1.52 | 0.03 | 3.69 | HMDB60372 | C04675 |
| 14 | Phosphatidylcholine | 14.91 | 956.80 | 1.29 | 0.03 | 3.66 | HMDB08783 | C00157 |
| 15 | Prephenate (PRE) | 14.91 | 957.22 | 1.22 | 0.04 | 3.35 | HMDB12283 | C00254 |
| 16 | 3-Oxooctanoyl-CoA | 5.90 | 908.19 | 1.25 | 0.04 | 3.07 | HMDB03941 | C05267 |
| 17 | Ceramides (d18:1/24:1(15Z)) | 10.60 | 648.60 | 1.80 | <0.01 | 3.04 | HMDB04953 | C00195 |
| 18 | Phosphatidyl ethanolamine (PE(24:1(15Z)/24:1(15Z))) | 14.43 | 912.76 | 1.02 | 0.04 | 2.95 | HMDB09773 | C00350 |
| 19 | Galactosylceramide (d18:1/24:0) | 14.92 | 974.71 | 1.31 | 0.04 | 2.94 | HMDB04840 | C06126 |
| 20 | Lactosylceramide (d18:1/24:0) | 14.69 | 974.70 | 1.55 | 0.04 | 2.94 | HMDB11595 | C01290 |
| 21 | Sphingomyelin (d18:0/12:0) | 11.03 | 651.50 | 1.47 | 0.01 | 2.70 | HMDB12084 | C00550 |
| 22 | Thioguanine | 14.91 | 955.89 | 1.19 | 0.04 | 2.66 | HMDB14496 | C07648 |
| 23 | Hemin | 11.04 | 652.20 | 1.66 | 0.02 | 2.46 | HMDB00887 | C06767 |
| 24 | Lysophosphatidylcholine (LysoPC) | 5.44 | 518.32 | 1.10 | 0.02 | 2.01 | HMDB10387 | C04230 |
| 25 | Itaconyl-CoA | 14.05 | 880.10 | 1.49 | 0 | 1.89 | HMDB03377 | C00531 |
| 26 | 7-Methyl-3-oxo-6-octenoyl-CoA | 14.78 | 920.18 | 1.42 | 0.03 | 0.10 | HMDB60421 | C16466 |
| 27 | Dolichyl β-D-glucosyl phosphate | 5.45 | 467.26 | 1.38 | 0.04 | 0.03 | HMDB01054 | C01246 |
| 28 | Citicoline | 5.46 | 489.07 | 1.41 | 0.04 | 0.01 | HMDB01413 | C00307 |

**Table S11** Significant metabolic pathways of GDM differential metabolites.

| Pathway Name | Total | Hits | Raw p | -Log(P) | Holm adjust | FDR | Impact |
| --- | --- | --- | --- | --- | --- | --- | --- |
| Glycerophospholipid metabolism | 39 | 5 | 0.00002 | 10.89900 | 0.00146 | 0.00074 | 0.37490 |
| Sphingolipid metabolism | 25 | 5 | 1.85E-06 | 13.19800 | 0.00015 | 0.00015 | 0.35384 |

**Table S12** Topological parameters of metabolic network of GDM differential metabolites

| Parameter | Parameter Values |  | Parameter | Parameter Values |
| --- | --- | --- | --- | --- |
| Node number | 188 |  | Clustering Coefficient | 0 |
| Edge number | 491 |  | Network density | 0.045 |
| Average number of Neighbors | 5.925 |  | Network isomerism | 1.103 |
| Network diameter | 9 |  | Network Centralization | 0.254 |
| Network radius | 5 |  | Connected Component | 6 |
| Characteristic path length | 3.861 |  | — | — |

**Table S13** Key target genes in GDM-associated differential metabolites and gene interaction network

| Gene | Degree centrality  (X =5) | Betweenness centrality  (X =0.041) | Closeness centrality (X =0.165) |
| --- | --- | --- | --- |
| PLD1 | 8 | 0.097 | 0.373 |
| PLD2 | 8 | 0.097 | 0.373 |
| EHHADH | 6 | 0.150 | 0.548 |
| HADHA | 6 | 0.150 | 0.548 |

**Table S14** The relative quality of circRNA in GDM pregnant women and controls

| Alias | Relative mass ratio (GDM /Non-GDM) | *P* |
| --- | --- | --- |
| hsa_circ_0042852 | 0.23 | 0.06 |
| hsa_circ_0004001 | 0.27 | 0.07 |
| hsa_circ_0006936 | 0.33 | 0.12 |
| hsa_circ_0001946 | 1.14 | 0.83 |
| hsa_circ_0000154 | 1.14 | 0.80 |
| hsa_circ_0006732 | 1.31 | 0.02 |
| hsa_circ_0001016 | 1.15 | 0.84 |
| hsa_circ_0001439 | 3.22 | 0.03 |

Table S15 Association of GDM-associatd gut genus and GDM-associatd differential metabolites

| GDM-associatd differential metabolites | GDM-associatd bacterial genus | r | *P* |
| --- | --- | --- | --- |
| Sphinganine 1-phosphate | Romboutsia | 0.29 | 0.02 |
| Hemin | Angelakisella | -0.34 | <0.01 |
| Citicoline | Raoultibacter | 0.37 | <0.01 |
| PA(16:0/16:0) | Anaerofustis | 0.31 | 0.02 |
| PE(24:1(15Z)/22:0) | Fusicatenibacter | -0.36 | <0.01 |
| PE(24:1(15Z)/24:1(15Z)) | Lactobacillus | 0.27 | 0.04 |
| Phosphatidylcholine | Fusicatenibacter | -0.35 | <0.01 |
| Glycochenodeoxycholic acid 3-glucuronide | Phascolarctobacterium | -0.34 | <0.01 |
| Sphingomyelin (d18:0/12:0) | Angelakisella | -0.32 | 0.01 |
| Sphingomyelin (d18:0/26:1(17Z)) | Catabacter | 0.35 | <0.01 |
| 2-(9Z,12Z-octadecadienoyl)-1-(1Z-hexadecenyl)-sn-glycero-3-phosphate | Turicibacter | -0.33 | <0.01 |
|  | Fusicatenibacter | -0.40 | <0.01 |
| PIP(16:0/20:1(11Z)) | Fusicatenibacter | -0.30 | 0.02 |
| 3-Oxooctanoyl-CoA | Lactobacillus | 0.28 | 0.04 |
|  | Bacteroides_pectinophilus_group | 0.27 | 0.04 |
| 7-Methyl-3-oxo-6-octenoyl-CoA | g_Oscillospirales_UCG-010 | 0.31 | 0.02 |
| (S)-Hydroxydecanoyl-CoA | Turicibacter | 0.26 | 0.04 |
|  | Fusicatenibacter | 0.28 | 0.03 |
|  | Phascolarctobacterium | 0.29 | 0.03 |
|  | Escherichia-Shigella | -0.30 | 0.02 |
| TG | Fusicatenibacter | -0.26 | 0.04 |
| PRE | Fusicatenibacter | -0.32 | 0.01 |

PA: Phosphatidic acid , PE; Phosphatidylethanolamine，TG:Triglycerides，PIP: Piperidine,

PRE: Prephenate

Table S16 Correlation between bacterial genera and differentially expressed circRNAs in GDM

| Differential expression circRNA | bacterial genus | r | *P* |
| --- | --- | --- | --- |
| hsa_circ_0042852 | Catabacter | -0.75 | <0.01 |
|  | Angelakisella | -0.59 | 0.04 |
|  | Escherichia-Shigella | 0.66 | 0.02 |
| hsa_circ_0004001 | Catabacter | -0.75 | <0.01 |
|  | Angelakisella | -0.59 | 0.04 |
|  | Romboutsia | 0.60 | 0.04 |
|  | Escherichia-Shigella | 0.61 | 0.04 |
| hsa_circ_0006936 | Catabacter | -0.75 | <0.01 |
|  | Angelakisella | -0.59 | 0.04 |
|  | Ruminococcaceae_UBA1819 | -0.63 | 0.03 |
| hsa_circ_0001439 | Catabacter | -0.75 | <0.01 |
|  | Angelakisella | -0.59 | 0.04 |
|  | Ruminococcaceae_UBA1819 | -0.80 | <0.01 |
|  | Escherichia-Shigella | 0.75 | <0.01 |
| hsa_circ_0006732 | Angelakisella | -0.65 | 0.02 |
|  | Ruminococcaceae_UBA1819 | -0.59 | 0.04 |
| hsa_circ_0000154 | Catabacter | -0.75 | <0.01 |
|  | Angelakisella | -0.59 | 0.04 |
|  | Escherichia-Shigella | 0.64 | 0.02 |
| hsa_circ_0001016 | Fusicatenibacter | 0.65 | 0.02 |
|  | g_Oscillospirales_UCG-010 | 0.65 | 0.02 |
