# Supplementary material for: Longitudinal associations between PM2.5 with gestational diabetes mellitus mediated by gut microbiome and potential mechanism: based on a prospective pregnant women cohort in China
Source: Front Cell Infect Microbiol. 2026 Feb 27;16:1749504. doi: 10.3389/fcimb.2026.1749504 (PMC12982432; doi:10.3389/fcimb.2026.1749504)
Supplement: Supplementary file 2 [file Table2.docx]

**Supplementary figs**


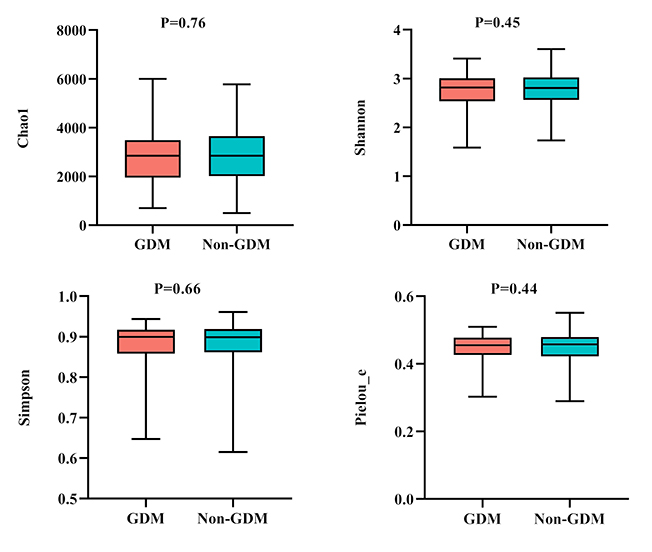


**Fig S1** Comparison of gut microbiota α diversity between GDM pregnant women and controls


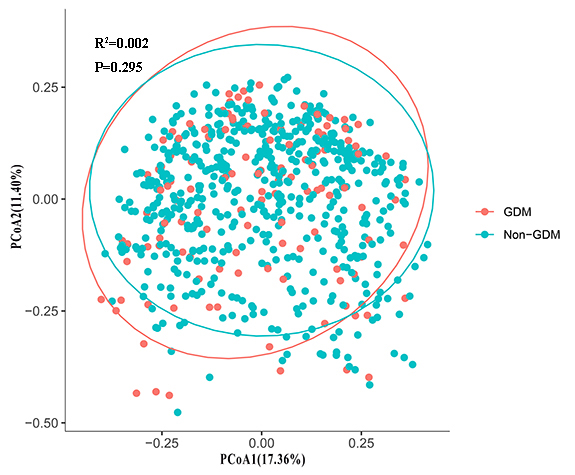


**Fig S2** PCoA analysis of gut microbiota gut microbiota β diversity between pregnant women with and controls
